# Differential Occupancy and Regulatory Interactions of KDM6A in Bladder Cell Lines

**DOI:** 10.3390/cells12060836

**Published:** 2023-03-08

**Authors:** Gülden Özden-Yılmaz, Busra Savas, Ahmet Bursalı, Aleyna Eray, Alirıza Arıbaş, Serif Senturk, Ezgi Karaca, Gökhan Karakülah, Serap Erkek-Ozhan

**Affiliations:** 1 Izmir Biomedicine and Genome Center, Inciralti, 35340 Izmir, Turkey; 2 Izmir International Biomedicine and Genome Institute, Dokuz Eylül University, Inciralti, 35340 Izmir, Turkey

**Keywords:** KDM6A, bladder cancer, chromatin regulation, protein–protein interactions, structural modeling

## Abstract

Epigenetic deregulation is a critical theme which needs further investigation in bladder cancer research. One of the most highly mutated genes in bladder cancer is *KDM6A*, which functions as an H3K27 demethylase and is one of the MLL3/4 complexes. To decipher the role of *KDM6A* in normal versus tumor settings, we identified the genomic landscape of *KDM6A* in normal, immortalized, and cancerous bladder cells. Our results showed differential *KDM6A* occupancy in the genes involved in cell differentiation, chromatin organization, and Notch signaling depending on the cell type and the mutation status of *KDM6A*. Transcription factor motif analysis revealed *HES1* to be enriched at *KDM6A* peaks identified in the T24 bladder cancer cell line; moreover, it has a truncating mutation in *KDM6A* and lacks a demethylase domain. Our co-immunoprecipitation experiments revealed *TLE* co-repressors and *HES1* as potential truncated and wild-type *KDM6A* interactors. With the aid of structural modeling, we explored how truncated *KDM6A* could interact with *TLE* and *HES1*, as well as *RUNX* and *HHEX* transcription factors. These structures provide a solid means of studying the functions of *KDM6A* independently of its demethylase activity. Collectively, our work provides important contributions to the understanding of *KDM6A* malfunction in bladder cancer.

## 1. Introduction

Chromatin deregulation is regarded as one of the hallmarks of cancer; it is reflected at multiple different levels and affects many processes that drive tumorigenesis [1]. Malfunction in epigenetic regulatory mechanisms is especially noteworthy in bladder cancer considering the prominently high rate of mutations in chromatin-modifier genes [2], which underscores the importance of studying epigenetic regulation in this cancer type.

Bladder cancer is one of the most common and lethal diseases worldwide. Each year, there are 430,000 cases of patients diagnosed with bladder cancer, 170,000 of which result in death [3]. In total, 75–80% of bladder cancers show non-muscle-invasive characteristics, while the remaining are muscle-invasive [2]. Despite recent genomic and molecular characterizations, cisplatin-based chemotherapy and radiotherapy-based approaches still remain the main treatment options for this cancer type [4]. Thus, within the context of high-chromatin-modifier mutations in this cancer [2], the identification of epigenetic signatures implicated in pathogenesis of this disease is crucial. 

Among the chromatin-modifier mutations observed in bladder cancer, a few of them are especially high, including *KMT2D* (28%), *KDM6A* (26%), and *ARID1A* (25%), which are observed in muscle-invasive bladder cancer [2]. Mutations identified in *KDM6A* in bladder cancer have loss-of-function characteristic [5], and there are many mutations identified within the intrinsically disordered region (IDR) of *KDM6A*, with one major mutation hotspot at position Q555 [5]. In this study, our focus is on identifying the differential function of *KDM6A* in normal, immortalized, and cancerous bladder cell lines. *KDM6A* is an H3K27 (lysine27 on histone H3) demethylase [6,7] that functions as a crucial chromatin modifier required for various developmental processes and for cellular differentiation [8]. Importantly, *KDM6A* is mutated in Kabuki syndrome, leading to facial abnormalities and cognitive dysfunction [8]. *KDM6A* is also required for embryonic development [9] and is involved in the resolution of bivalent chromatin domains [10]. *KDM6A*’s role in the regulation of *Hox* genes is well characterized [6]. In addition, *KDM6A* function has been identified to be important for heart development [11] and hematopoiesis [12]. On a biochemical level, *KDM6A* has been shown to be associated with the evolutionarily conserved *MLL3*/*MLL4* COMPASS complex, which is involved in the methylation of H3K4 and active chromatin organization in several cell lines [7,8]. *KDM6A* is directly bound to the C-terminal of *MLL3*/*MLL4* [13]. This indicates that *KDM6A* may play a role in regulating the catalytic activity of *MLL3*/*4*. 

*KDM6A* is mutated in 26% of muscle-invasive bladder cancer cases, whereas its mutation frequency is higher in non-muscle-invasive bladder cancer (47.6%) (cBioPortal TCGA2017, MSK2017). However, the exact molecular mechanisms underlying the functions of *KDM6A* in a normal cellular setting and its promotion of tumorigenesis are currently open questions that need to be addressed in detail. Expanding on this, in this study, we generated KDM6A ChIP-seq data for one normal urothelial cell line (BdEC), one immortalized non-tumorigenic urothelial cell line (SV-HUC-1), and one urothelial cancer cell line (T24). The hypotheses we developed from the ChIP-seq data were further explored using biochemical assays and structural modeling. As a result, we show that *KDM6A* has a differential occupancy profile in the analyzed cell lines, and it shows distinct behavior when it is mutated. We also identify the involvement of *KDM6A* in the wide-spread regulation of Notch signaling in bladder cell lines. Furthermore, we structurally model the interaction of truncated *KDM6A* with repressors and co-repressors. Collectively, these results provide clear insights into previously uncovered roles of *KDM6A* in gene regulation. 

## 2. Materials and Methods

### 2.1. Experimental Procedures

#### 2.1.1. Cell Culture

The BdEC (primary bladder epithelial cells) (ATCC^®^ PCS-420-010™), SV-HUC-1 (immortalized uroepithelial cells) (ATCC^®^ CRL-9520™), T24 (urinary bladder transitional cell carcinoma) (ATCC^®^ HTB-4™) and HEK293T cell lines were used for the experiments. The BdEC cell line was grown in the appropriate medium (ATCC^®^ PCS-440-030™) and supplemented with the Prostate Epithelial Cell Growth Kit (ATCC PCS-440-040), as recommended by the ATCC, and Primocin (Invivogen). SV-HUC-1 was grown with RPMI-1640, and T24 and HEK293T were grown with DMEM, supplemented with 10% FBS and 1% penicillin. The cells were maintained in a humidified incubator at 37 °C and 5% CO_2_. 

#### 2.1.2. Chromatin Immunoprecipitation 

The chromatin immunoprecipitation assays were performed by following the protocol of Weber et al., 2007 [14]. Briefly, the cells were fixed with 1% formaldehyde for 15 min when they reached 70–80% confluence. A final concentration of 0.125 M glycine was used to prevent crosslinking. After the pellet was lysed with lysis buffer, the chromatin was fragmented using a S220 Covaris Ultrasound Sonicator. With the intent of determining the optimum sonication for 200–500 bp fragmented DNA, a 50 µL input sample was taken from the lysate. The rest of the chromatin was used for the immunoprecipitation (IP). For IP, the chromatin was first pre-cleared using Dynabeads (Invitrogen, Waltham, MA, USA, 11203D) blocked with BSA and tRNA, and then, bound with 5 µL KDM6A antibody (Cell Signaling Technology, Danvers, MA, USA, 33510) overnight at 4 °C. The next day, the chromatin–antibody complex was bound with pre-blocked Dynabeads for 3 h at 4 °C, and after the washing steps, the chromatin was eluted. For both the input and IP DNA, following the RNase and proteinase K treatment, the samples were incubated overnight at 65 °C for reversal of the crosslink. The DNA was eluted using a DNA Clean & Concentrator kit (Zymo Research, Orange, CA, USA, D4034). 

#### 2.1.3. ChIP-Seq

For the T24, BdEC, and SV-HUC-1 cell lines, ChIP and input DNA were sent to the EMBL GeneCore facility for library preparation and sequencing. A NEBNext DNA Ultra II kit was used for library preparation. The libraries were sequenced on a Nextseq 500 platform using 75bp SE high-output mode. 

#### 2.1.4. RT-qPCR

Total RNAs were isolated using an MN Nucleospin RNA kit from each cell line. A total of 0.25 µg RNA was used for cDNA conversion using a Maxima First Strand cDNA Synthesis Kit. PCR was performed using the Applied Biosystems 7500 Fast Real-Time machine with a Roche FastStart Essential DNA Green Master (SYBR, Roche, Penzberg, Germany) kit for the selected genes. The experiments were performed as three technical replicates.

#### 2.1.5. ChIP-qPCR

After performing the *KDM6A* ChIP experiments for the T24, SV-HUC-1, and BdEC cell lines, qPCR was performed using the Roche FastStart Essential DNA Green Master (SYBR) kit on an Applied Biosystems 7500 Fast Real-Time machine, and the results are presented as % input IP. The experiments were performed as three technical replicates. The ChIP-qPCR results were calculated using the ΔΔct method. ChIP Ct values were normalized to the input. 

#### 2.1.6. Primer Design

The primers for RT-qPCR and ChIP-qPCR were designed using the primer designing tool Primer-Blast (NCBI). A list of all the primers can be found in Table S3.

#### 2.1.7. Western Blotting 

The cells were washed with ice-cold PBS and scraped with 1× RIPA buffer supplemented with a protease inhibitor cocktail. After vortexing and incubating for 10 min on ice three times, the lysates were centrifuged at max speed at 4 °C for 20 min and the supernatants were collected. A Pierce BCA Protein Assay Kit (Thermo Fisher Scientific, Rockford, IL, USA, 23225) was used to determine the protein concentrations. A total of 30 µg proteins were loaded onto 8% acrylamide:bis-acrylamide gel. The gel was run at 90 V for 30 min and at 120 V for one and a half hours. Gel transfer was performed at 350 mA for one and a half hours in a box full of ice. Non-fat dried milk powder was used for blocking the membrane for one hour at room temperature. *KDM6A* (CST 33510), *HES1* (CST 11988), *TLE1/2/3/4* (CST 4681S), and b-actin (CST 3700) primary antibodies were used with a 1/1000 dilution, and CST 5151S and LICOR 926-68072 secondary antibodies were used with a 1/30,000 dilution. Nitrocellulose membrane was incubated overnight at 4 °C with the *KDM6A* primary antibody, and for one and a half hours at room temperature with the b-actin antibody. Secondary antibody incubations were performed for one hour at room temperature. The membrane was imaged using the Li-COR ODYSSEY Clx machine (LiCOR, Lincoln, NE, USA) in auto mode. 

#### 2.1.8. Identification of KDM6A Mutation

DNA was extracted from the cell lines BdEC, T24, and SV-HUC-1 using a ZymoResearch (Irvine, CA, USA) Quick DNA Miniprep Plus kit (D4068). Mutation analysis was performed using the Archer VariantPlex Myeloid Panel (Diagnostica Longwood, Zaragoza, Spain), an NGS panel containing the *KDM6A* gene. Mutation calling was performed using ArcherDx Analysis (Version 6.2.7) with default settings using the Gen-ERA NGS service.

#### 2.1.9. Plasmid Constructs and Transfection 

To generate the *FLAG*-*KDM6A* construct, a pCMV_HA_UTX (Addgene, Plasmid #24168, Watertown, MA, USA) vector was digested for 2 h at 37 °C using Kpn1 and Not1 restriction enzymes, and the *KDM6A* gene was removed from this vector. In order to add a FLAG-tag to *KDM6A*, 2-step PCR was set up using the Q5 polymerase (M0494S, Neb, Ipswich, MA, USA) with designed primers (see primer list, Table S3). The PCR product was cut from the agarose gel and isolated using NucleoSpin^®^ Gel and a PCR Clean-up kit (MN, 740609, Düren, Germany). The digested FLAG-tag KDM6A gene and the digested pcDNA3.1(+)/myc-HisA (Invitrogen) vector were ligated for 2 h at room temperature using T4 Ligase (Neb, M0202S) in accordance with the Neb Ligation Calculator (https://nebiocalculator.neb.com/#!/ligation, accessed on 1 April 2022). The ligation product (pcDNA3.1_FLAG_KDM6A) was transformed into an *E. coli* 10beta bacterial strain. Growing colonies were isolated using the plasmid isolation kit (MN, 740588) and validation was performed via Sanger sequencing. A total of 80% confluent 10cm plate HEK293T cells were transfected with 7.5 µg plasmid DNA using Lipofectamine 3000 (Invitrogen, L3000015) reagent. The medium was changed 24 h after transfection. To generate the *KDM6A* truncated mutation like in the T24 cell line, a pcdna3.1_FLAG-KDM6A plasmid was digested for 2 h at 37 °C using Kpn1 and Not1 restriction enzymes. After being run on 1% agarose gel at 100 V for 45 min, the FLAG-KDM6A band was isolated using NucleoSpin Gel and the PCR Clean-up kit (MN, Düren, Germany) from the gel. two-step PCR was set up using the Q5 polymerase with designed primers for E895* mutation (see primer list, Table S3). After this, the same steps as for the generation of the pcDNA3.1_FLAG_KDM6A plasmid were followed.

#### 2.1.10. Co-IP

Proteins from untransfected and pcDNA3.1_FLAG_KDM6A or pcDNA3.1_FLAG_E895*mutant_KDM6A transfected HEK293T cells (48 h post transfection) were isolated using 300 µL lysis buffer (50 mM Tris-HCl pH:7.4, 150 mM NaCl, 1 mM EDTA, 1% TritonX-100, Protease Inhibitor Cocktail (Roche, Indianapolis, IN, USA)). For input controls 20 µL untransfected and transfected HEK293T cell lysates were used. Remaining proteins were bound overnight at 4 °C on the rotator with Flag affinity gel (Sigma, A2220, St. Louis, MO, USA) in accordance with the manufacturer’s instructions. After the incubation, IP proteins were centrifuged, and pellets were washed with TBS three times. Proteins for both input and IP fractions were boiled with SDS Sample Buffer (62.5 nM Tris-HCl pH:6.8, 2%SDS, 10% (*v*/*v*) Glycerol and 0.002% Bromophenol Blue) at 95 °C for 3 min and analyzed by Western blotting. *KDM6A* (CST 33510), *HES1* (CST 11988) and *TLE1/2/3/4* (CST 4681S) antibodies were used for Western blotting.

### 2.2. Data Analysis and Structural Modeling Procedures

All the bioinformatics analyses were performed at IBG Bioinformatics Platform.

#### 2.2.1. Alignment and Processing of Next-Generation Sequencing Data

The ChIP-seq data were aligned to the human genome (hg38) using the nf-core/chip-seq pipeline (https://nf-co.re/chipseq/1.2.1, accessed on 5 February 2021). Using this pipeline, *KDM6A* ChIP peaks were called, peak annotation was performed, and *KDM6A* signals on peaks were quantified in CPM. 

#### 2.2.2. Peak Calling and Peak Overlap 

*KDM6A* peak calling was performed using MACS2 (embedded in the nf-core chip-seq pipeline) using the parameters: *p* < 10^−5^ and FDR < 0.1 and a filter with the narrow option. For the set of peaks called for the T24, SV-HUC-1, and BdEC cell lines, the overlap among the peaks was checked using the “VennDiagram package” in R. 

#### 2.2.3. Clustering of KDM6A Peaks 

Only the peaks which were located within a ±2 kb distance of the transcriptional start site (TSS) of genes and that had an fc value > 3 (value coming from MACS) were used for the clustering analysis. Before clustering, CPM counts were normalized for the library sizes, and the log2 of the normalized values were used for plotting. Hierarchical clustering was performed using the “pheatmap package” (https://cran.r-project.org/web/packages/pheatmap/index.html, accessed on 15 March 2021) in R. Clusters were determined and visualized using the cutree = 4 option in the package. 

#### 2.2.4. Analysis of T24 *KDM6A* Peaks 

The TSS ±2 kb filtered peaks in the Venn Diagram were used for the analysis. Peaks were ranked according to their CPM normalized log2 values for each cluster. The pheatmap R package was used for visualization.

#### 2.2.5. Transcription Factor Motif Finding 

Transcription factor motif finding for different *KDM6A* clusters was performed using the findMotifsGenome.pl command of HOMER (http://homer.ucsd.edu/homer/motif/, accessed on 17 March 2021), with a size parameter of 300, using genomic positions. 

#### 2.2.6. Gene Ontology Analysis

Gene Ontology (GO) term analysis was performed using ConsensusPathDB (http://cpdb.molgen.mpg.de/, accessed on 22 March 2021) with a GO Level of 3 and biological process (BP) options. The top 10 terms for ‘IH-MULTI’, ‘normal’, and ‘normal-immortal’ clusters were identified, and for those terms, GO Level 3 was visualized using the dotplot function of the ggplot2 package (https://cran.r-project.org/web/packages/ggplot2/index.html, accessed on 5 August 2021). Only the terms with a qval < 0.01 filter were visualized.

#### 2.2.7. Data Visualization

ChIP-seq signal data visualization was performed using the Bioconductor Gviz package (https://bioconductor.org/packages/release/bioc/html/Gviz.html, accessed on 1 June 2021). “BW” files were obtained from CPM-normalized BAM and BAI files using deeptools “BamCoverage” functions’ default parameters. Visualization was performed with “BW” files using the ‘horizon’ type of GViz package. GENCODE hg38 Comprehensive Gene Annotation Version 30 data were used as the GeneRegionTrack for the annotation. 

#### 2.2.8. String Protein Interaction Data Visualization

For protein interaction data visualization, Cytoscape (version 3.8.2)’s default style was used with String data.

#### 2.2.9. *HHEX* and *HES1* Gene Expression-Related Associations

To obtain correlation heatmaps between the expressions of *HHEX* and *HES1*, primary bladder cancer Hiseq data from TCGA BLCA 2017 (*n* = 426) and bladder cancer cell line RNAseq data (*n* = 25) from CCLE (https://depmap.org/portal/download/all/(CCLE_RNAseq_genes_rpkm_20180929.gct, accessed on 17 December 2021) were used. Pearson correlation coefficients for the correlation of the expression of *HHEX* and *HES1* and the genes associated with the term ‘regulation of the developmental process’ within the normal-immortal cluster (*n* = 34) were calculated using RPKM-normalized log2 expression values. The ggplot2 (version 3.3.5) and pheatmap (version 1.10.12) packages in R were used for visualization. 

#### 2.2.10. Survival Analysis

To generate Kaplan–Meier graphs showing patients survival times according to different expression levels of *HHEX* and *HES1*, gene expression RNAseq IlluminaHiSeq* (*n* = 426) data and phenotype-curated survival data (*n* = 436) belonging to a TCGA Bladder Cancer (BLCA) cohort from Xena Browser Datasets [15] was used. Patients with survival data of OS.time > 120 (*n* = 382) were included in the Kaplan–Meier graphs. Low/high expression groups were formed by calculating the median values for *HES1* and *HHEX* genes separately. Kaplan–Meier graphs were created using a log-rank test according to OS.time values. The coin (https://cran.r-project.org/web/packages/coin/index.html, accessed on 4 January 2022) (version 1.4-2) and survival (https://cran.r-project.org/web/packages/survival/index.html, accessed on 4 January 2022) (version 3.2-7) R packages were used for the analysis. 

#### 2.2.11. Comparison of T24 *KDM6A* Peaks with Published Data

To compare the *KDM6A* peak profile in the T24 cell line to a wild-type *KDM6A* peak profile called in a bladder cancer cell line, published WT *KDM6A*-expressing UMUC-1 bladder cancer cell line’s narrowpeak data (Barrows et al., GSE157091) were used. Peak overlaps were performed using the ‘findOverlaps’ function and the rtracklayer (https://bioconductor.org/packages/release/bioc/html/rtracklayer.html, accessed on 15 January 2023) and Genomic Ranges R packages (https://bioconductor.org/packages/release/bioc/html/GenomicRanges.html, accessed on 15 January 2023), and subsequently visualized using a Venn diagram. A snapshot showing *KDM6A* occupancy at *HES1* and *TLE3* loci in wild-type *KDM6A*-expressing UMUC-1 cells was created using the IGV genome browser (https://software.broadinstitute.org/software/igv/, accessed on 15 January 2023). 

#### 2.2.12. Structural Modeling

To support our in vitro findings, we modeled potentially interacting protein pairs linked to the truncated KDM6A interaction network, i.e., *TLE1*, *HES1*, *RUNX1*, and *RUNX2*. For this, first, we retrieved the available relevant PDB structures from the Protein Data Bank [16]. We also gathered the structures of homologous protein complex systems that would help us model the unknown protein complexes. For the proteins with unknown structures, we used the deep learning-based protein modeling tool AlphaFold2 (AF2) [17]. 

##### *KDM6A*:*TLE1* Interaction Modeling

*KDM6A* folds into the tetratricopeptide repeat (TPR) and jumanji family (JmjC) domains. These two domains are connected by long-disordered regions between the 437th and 1079th residues. Resolved *KDM6A* structures start from the 877th residue; therefore, they lack the N-terminal TPR domain (PDB IDs: 3AVR, 3AVS, 6FUK, and 6FUL) [18,19] The full-length *TLE1* is a 770-amino acid-long protein containing a glutamine-rich (Q), a glycine–proline-rich (GP), a nuclear localization signal-containing-central (CcN), a serine–proline-rich (SP), and a C-ter WD40 repeat domain (WDR). Among these domains, only the Q and WDR domains were experimentally determined for the *TLE1* protein (PDB IDs: 1GXR, 2CE8, 2CE9, 4OM2, 4OM3, and 5MWJ) [20,21,22,23]. To model the *KDM6A*:*TLE1* complex, we focused on the TPR and Q domains. The TPR motif is formed of 34-amino acid-long alpha-helix pair repeats and possesses a helical curvature. The structure of the Q domain is represented in the 4OM2 and 4OM3 PDB entries [22]. In these structures, *TLE1*-Q forms a coiled-coil dimer through the interaction of 70-amino acid-long alpha helices belonging to different *TLE1*s.

For the interaction modeling, initially, we modeled the TPR domain with AF2 using default parameters [17]. Then, we modeled *KDM6A*-*TPR*:*TLE1*-Q with an AlphaFold-multimer (data not shown here). Assessment of the AlphaFold models was carried out by analyzing: (i) pLDDT scores showing the predicted confidence level of each residue, (ii) predicted aligned error (PAE) graphs indicating the predicted position errors of each residue, and (iii) predicted TM (PTM) and interface predicted TM (iPTM) scores representing the overall accuracy of the complex and interface, respectively. 

To generate good-quality models, we further searched the PDB Data Bank to find complexes containing TPR and coiled-coil interactions. The 6EJN PDB structure was chosen as a reference here [24]. In this structure, the leucine zipper-like domain of JNK-interacting protein 3 (JIP3, a cargo protein) is found in a coiled-coil dimer form. The cargo interacts with two different mouse kinesin light chain 2 proteins (KLC2, a transport protein) through their first TPR motifs. The overall *KLC2*:*JIP3* structure has a stoichiometry of 2:2. As this structure provides the binding pose for TPR and the coiled-coil interaction, we generated our *KDM6A*-*TPR*:*TLE1*-Q model based on the 6EJN complex. To build the targeted complex, we used the best-ranked *KDM6A*-TPR AF2 model. The coiled-coil TLE1 dimer was retrieved from the 4OM3 PDB entry [22]. We used the flexible structural alignment server FATCAT to superimpose the first three TPRs of *KLC2* and *KDM6A* [25]. The obtained superimposed models were further used during structural fitting via PyMOL along with coiled-coil alignments, leading to our final model (The PyMOL Molecular Graphics System, Version 2.0, Schrödinger, LLC, 1 December 2022).

##### *TLE1*:*HES1* Interaction Modeling

*HES1* contains basic helix–loop–helix (bHLH) and orange domains and a Trp-Arg-Pro-Trp (WRPW) motif located at its C-terminal end. The WRPW motif is required for binding to Groucho/TLE family members. The PDB entry 2CE9 contains an interaction between the WRPW peptide and the Human Groucho/TLE WDR domain. In this structure, the WDR domain interacts with the WRPW peptide in a 2:1 fashion. This *TLE1*:*HES1* complex has been further refined using the HADDOCK 2.4 webserver [26,27]. HADDOCK is a user-friendly tool for performing biomolecular docking. After carrying out docking protocols, it also performs short molecular dynamics simulations to refine the interface by enabling sidechain movements. When turning off the docking procedures, HADDOCK completes the missing atoms in the input structures and improves the interface. To preserve the binding mode, we only applied the refinement step with unambiguous distance restraints to keep the peptide at its binding position (see advanced refinement protocol of HADDOCK 2.4 for details) [28]. 

##### *TLE1*:*RUNX1/2* Interaction Modeling

*RUNX1* and *RUNX2*, hereinafter referred to as *RUNX* in the text, are characterized by an approx. 120-amino acid-long RUNT domain, which is required for DNA recognition. The C-ter Trp-Arg-Pro-Tyr (WRPY) motif is known to interact with *TLE1*-WDR. We modeled this complex by taking the 2CE9 PDB as a template. During modeling, we mutated the last amino acid in the WRPW peptide to a tyrosine to match the WRPY motif of *RUNX*, and refined it using HADDOCK2.4, as described above. 

##### *TLE1*:*HHEX* Modeling

The primary structure of *HHEX*, also known as the proline-rich homeodomain protein (PRH), consists of three domains: an N-terminal proline-rich (P-rich) domain, a central homeodomain that interacts with DNA, and a C-terminal acidic domain that regulates transcription activity. The 137-amino acid-long P-rich domain includes an EH1 motif (FxIxxIL) that provides an interaction surface for *TLE1*. From the Protein Data Bank, we retrieved the 2CE8 structure, showing the interaction of *TLE1*-WDR with the EH1 peptide (FSIDNIL) from human Goosecoid (GSC), another homeobox protein. To mimic the sequence of the EH1 motif in *HHEX* and *FYIEDIL*, we mutated the mismatched amino acids. Mutation and refinement procedures were performed as outlined above. 

Further, we used AlphaFold-multimer to model the interaction between *TLE1*-Q and *HHEX* [29]. AlphaFold-multimer allows for the prediction of multimeric protein complexes with a known stoichiometry. Here, our input sequences contained the N-terminal 131 and 98 amino acids of *TLE1* and *HHEX*, respectively. We modeled the *TLE1*:*HHEX* complex with a stoichiometry of 2:1. The default parameters were used for multimer modeling in our local machine. Assessment of the models was performed as described above. 

#### 2.2.13. Statistical Analysis

The R environment was used for all data analysis and plots. Kaplan–Meier graphs were created using a log-rank test according to OS.time values. ChIP-qPCR and gene expression data were visualized using GraphPad Prism 9. Pearson correlation coefficients were calculated to determine the correlation between gene expression values. For RT-qPCR’s significance scores, one-way ANOVA and Tukey’s multiple comparison test were used.

## 3. Results

### 3.1. KDM6A Mutation Status, Identification, and Classification of KDM6A Peaks in Bladder Cell Lines

**Figure 1:**
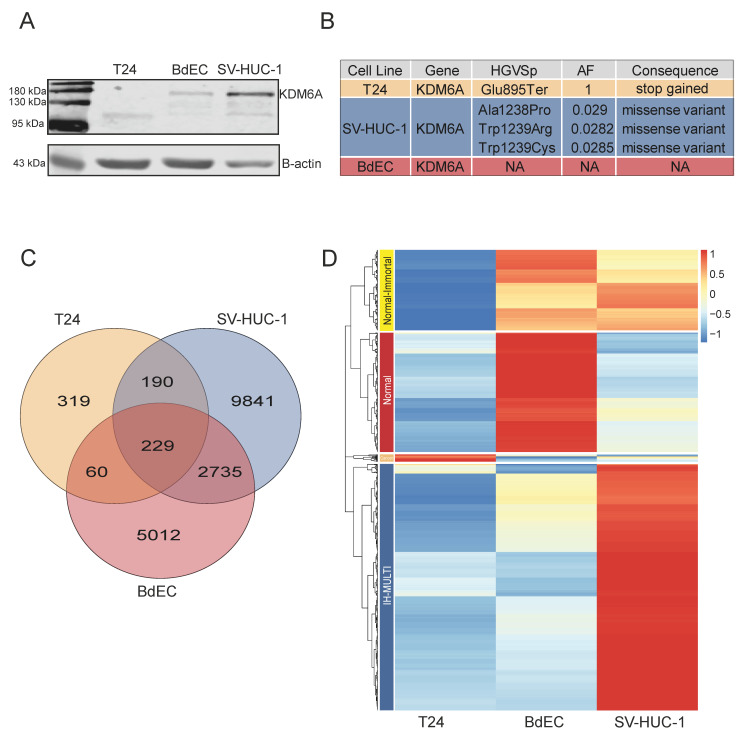
Occupancy and mutation profile of *KDM6A* in different bladder cell lines. (**A**) Western blot image displaying expression of *KDM6A* at protein level. (**B**) Image represents the details concerning depth, allele frequency, and alternative alleles related to mutation status of *KDM6A*. (**C**) Venn diagram showing the overlap among *KDM6A* peaks called in T24, SV-HUC-1, and BdEC cell lines. (**D**) Heatmap shows the hierarchical clustering of *KDM6A* peaks, resulting in 4 clusters: normal-immortal (*n* = 235), normal (*n* = 349), cancer (*n* = 21), and IH-MULTI (*n* = 721).

We performed *KDM6A* ChIP-seq for three different cell lines: BdEC (normal primary bladder epithelial cells), SV-HUC-1 (immortalized bladder epithelial cells), and T24 (the muscle-invasive bladder cancer cell line). Among the cell lines analyzed, T24 had a known stop-gain mutation (position: E895). Prior studies showed that this mutation was homozygous, and Western blot analysis revealed a protein product about 110 kDa in size [30]. Our own Western blot analysis also confirmed the presence of *KDM6A* in all three cell lines, and a truncated mutated product for the T24 cell line (Figure 1A). The mutant *KDM6A* observed for the T24 cell line lacked the histone demethylase domain. To be thorough about identifying the mutation status of the cell lines used in the study, we also determined the mutation status of *KDM6A* in all three cell lines ourselves. As expected, the T24 cell line had a homozygous truncating mutation at position 895 for *KDM6A*. The SV-HUC-1 cell line had missense mutations at positions 1238 and 1239 with allele frequencies of 0.029 and 0.0282, while BdEC was a wild type for *KDM6A* (Figure 1B). We considered the missense mutation observed for SV-HUC-1 to be a reflection of a clonal mosaic pattern and to have a negligible effect on chromatin binding.

We called *KDM6A* peaks in the three cell lines from our ChIP-seq data, resulting in 8050, 13,039, and 819 f peaks in the BdEC, SV-HUC-1, and T24 cell lines, respectively. A total of 16% of peaks were shared between the BdEC and SV-HUC-1 cell lines, whereas the total number of peaks called for the T24 cell lines was rather limited. Here, only 229 peaks were common among the all three cell lines used (Figure 1C). We then proceeded with the identification of *KDM6A* peaks with differential enrichment of the *KDM6A* signal. This analysis resulted in four distinct clusters, which we named ‘normal-immortalized shared’ (normal-immortal) (*n* = 235), ‘normal’ (*n* = 349), ‘cancer’ (*n* = 21), and ‘immortalized high-multiform in cancer and normal’ (IH-MULTI) (*n* = 721) (Figure 1D).

### 3.2. Characterization of Genomic Loci with Differential KDM6A Signals

**Figure 2:**
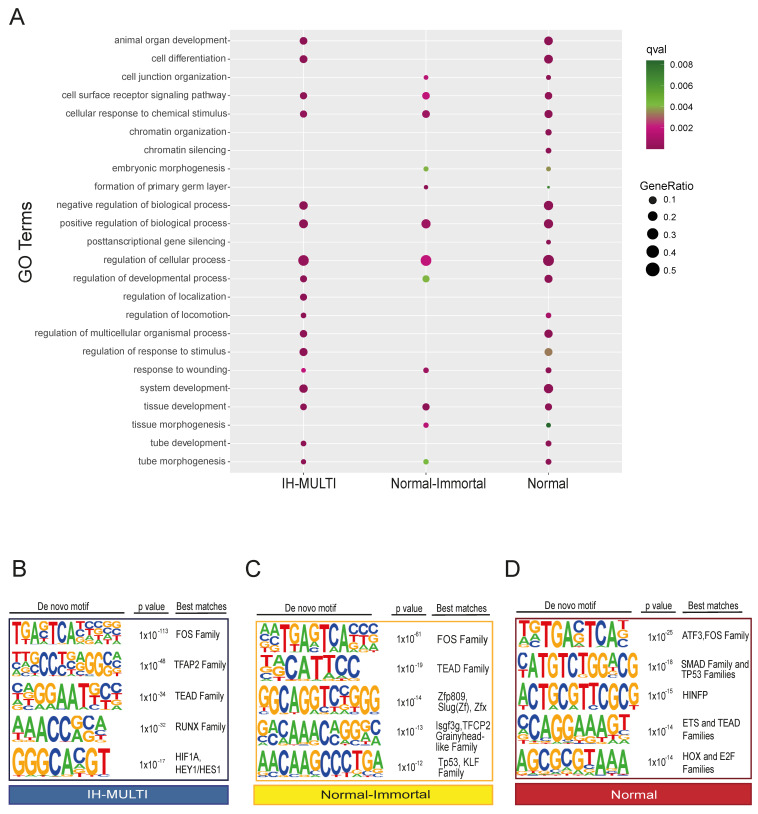
Functional analysis of differential KDM6A peak sets identified across the cell lines. (**A**) Dot plot image displaying the GO term analysis performed for the genes associated with different clusters. (**B**–**D**) Transcription factor motif analysis performed for IH-MULTI (**B**), normal-immortal (**C**), and normal (**D**) clusters. Up to 3 different transcription factors/transcription factor families are shown with the best motif matching scores.

After determining the genomic regions with differential *KDM6A* signals in the cell lines analyzed, we annotated them with functional features. First, we identified the genes associated with the peaks located in the clusters we defined (Figure 1D) (peaks located within a ±2kb distance of the transcriptional start sites (TSS); see Methods), and performed GO term enrichment analysis for the genes identified in different clusters. Genes associated with the ‘cancer’ cluster were not significantly enriched in the GO analysis. For the other three clusters, terms related to general developmental functions showed similar enrichment patterns for all three clusters. The ‘Normal’ and ‘normal-immortal’ clusters were similarly enriched for ‘cell junction organization’ (Figure 2A, Table S1). On the other hand, we identified ‘chromatin organization’-related terms to be significantly enriched only in the ‘normal’ cluster (Figure 2A). These genes, exceptionally, mainly consisted of replication-dependent histone genes (Figure S1A,B). In contrast, non-canonical histones did not have a *KDM6A* signal in any of the three cell lines (Figure S1C,D). 

Next, we performed transcription factor motif analysis for the peaks belonging to different clusters (Figure 1C). All clusters were similarly enriched for *FOS* and *TEAD* family transcription factor motifs (Figure 2B–D). We identified enrichment in several members of the zinc finger family, grainyhead-like family, and p53 transcription factor motifs at peaks located within the ‘normal-immortal’ cluster, while the *RUNX* and bHLH families of transcription factors (*HIF1A*/*HEY1*) were enriched at peaks within the ‘IH-MULTI’ cluster (Figure 2B,C). The ‘Normal’ cluster showed enrichment in the Smad family, p53, and *HINFP*, a transcription factor involved in transcriptional regulation of histone *H4* [31] (Figure 2D). As the identification of transcription motifs only does not directly provide information about the functional involvement of these factors, we checked the expression of several transcription factors with motifs enriched in these clusters, which might be relevant for bladder cancer biology. Combined with the motif finding results, these data (Figure S2) suggest that *KDM6A* might potentially cooperate with the *TP53* family and the *GRHL2* and *FOSL2* transcription factors to drive gene expression associated with epithelial characteristics; meanwhile, coordination with transcription factors such as *FOSL1*, previously described to be involved in the invasive characteristic of bladder cancer [32,33], might contribute to the gene expression programs that drive tumorigenesis.

### 3.3. KDM6A Occupies the Genes Involved in Notch Signaling

**Figure 3:**
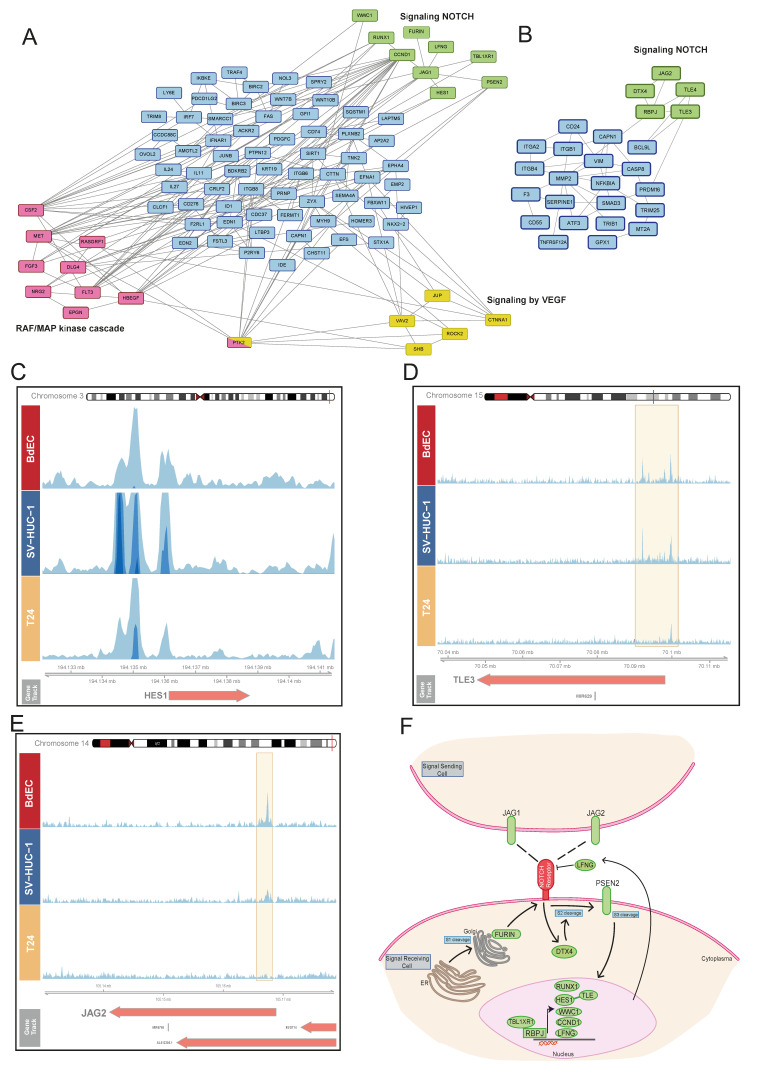
Genes regulated by *KDM6A* are implicated in notch signaling. (**A**,**B**) Analysis of the genes involved in cell surface receptor signaling for IH-MULTI (**A**) and normal-immortal clusters (**B**). Interaction data obtained from String was visualized using Cytoscape. (**C**–**E**) Snapshot images showing *KDM6A* signal in BdEC, SV-HUC-1, and T24 cell lines at *HES1* (**C**), *TLE3* (**D**), and *JAG2* (**E**) loci. (**F**) Manually created cartoon showing the genes involved in Notch signaling and associated with *KDM6A* peaks (**A**,**B**). Genes marked with *KDM6A* signal are highlighted in green.

To decipher the signaling pathways associated with *KDM6A* function, we analyzed the term ‘cell surface receptor signaling pathways’, which are similarly enriched for different clusters. The ‘IH-MULTI’ class showed enrichment in the Raf/MAP kinase cascade and VEGF signaling (Figure 3A). Surprisingly, genes involved in cell surface receptor signaling showed clustering of the genes associated with Notch signaling for both the ‘IH-MULTI’ and ‘normal-immortal’ clusters (Figure 3A,B). The ‘Normal’ class also had enrichment in Notch signaling. However, we discovered that these mostly consisted of histone genes, reflecting enrichment of the genes involved in chromatin organization in the ‘normal’ cluster (Figure S3). 

Notch signaling has a key role in normal urothelium development, and its deregulation is implicated in bladder tumorigenesis [34,35]. We identified a *KDM6A* signal at the promoter regions of several prominent Notch signaling-related genes, such as *HES1*, a transcriptional repressor, and *TLE3/4*, a transcriptional co-repressor, both critical for cell fate decisions [36]; *JAG1* and *JAG2*, ligands of the NOTCH receptor genes [37]; and *RBPJ*, a DNA binding protein which can complex with cleaved Notch to activate transcription [38] in all three cell lines with different signal intensities (Figure 3C–E and Figure S4A,B), covering all prominent components of Notch signaling (Figure 3F). Upon checking the expression of *KDM6A*-occupied Notch signaling genes in the analyzed cell lines, we identified that the occupancy level of *KDM6A* correlates with the expression level of the genes overall (Figure S4C), supporting the idea of a relationship between active gene expression and chromatin organization by *KDM6A*. However, it should be noted that relative levels of transcription factors and repressive chromatin remodeler complexes might be critical in the regulation of gene expression by *KDM6A*, as has been shown in the literature before [10,39]. Collectively, our results underscore the role of *KDM6A* in the regulation of Notch signaling and its potential deregulation in bladder cancer.

### 3.4. Truncated KDM6A Associates with Genes Deregulated in Cancer

**Figure 4:**
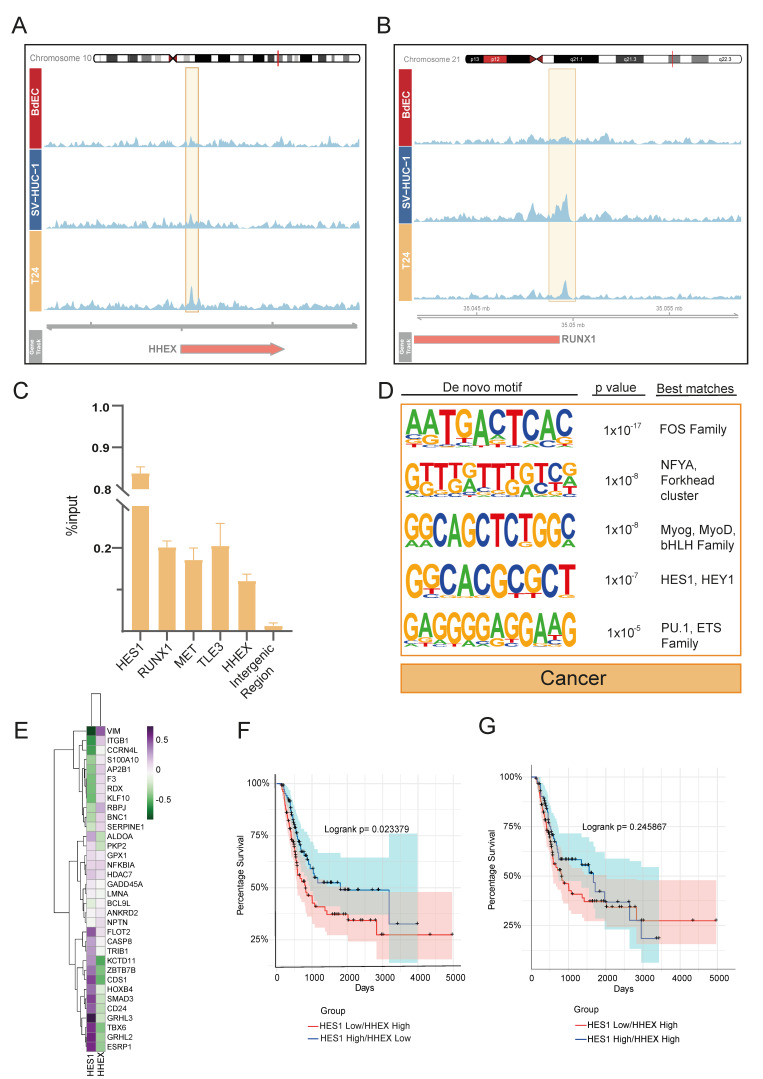
Genomic occupancy profile of truncated *KDM6A* in T24 bladder cancer cell line. (**A**,**B**) Snapshot images showing *KDM6A* signal in BdEC, SV-HUC-1, and T24 cell lines at *HHEX* (**A**) and *RUNX1* (**B**) loci. (**C**) ChIP-qPCR analysis of *KDM6A* enrichment in T24 cell line at selected loci. (**D**) Transcription factor motif enrichment analysis of T24 *KDM6A* peaks linked with genes. (**E**) Heatmap demonstrates the correlation between the expression of *HES1*, *HHEX*, and the genes involved in the regulation of developmental processes in primary bladder cancer. (**F**) Kaplan–Meier graph shows the overall survival of primary bladder cancer patients, grouped according to the expression status of *HES1* and *HHEX*. (**G**) Kaplan–Meier graph shows the overall survival of primary bladder cancer patients, grouped according to the expression status of HES1 with high ‘*HHEX*’ criteria.

We were able to find only a limited number of peaks annotated with a gene for the ‘cancer’ cluster (*n* = 20) (Figure 1D). Performing a GO enrichment analysis for this set of genes did not result in any significant GO term. However, as we have shown, the truncating mutation at position 895 resulted in a protein product, and this product still showed some chromatin binding profiles. Thus, we re-inspected all T24 *KDM6A* peaks within a ±2 kb distance from TSS (Figure 1C), independently of their cell line specificity (*n* = 50), (Table S2) to decipher more mechanistic insights which might be associated with truncated *KDM6A*. In relation to our initial clustering analysis (Figure 1D), most T24 *KDM6A* peaks were either located in the ‘IH-MULTI’ (42%) or ‘cancer’ (40%) clusters. The peaks with the highest signal intensity were also found in these respective clusters (Figure S5A). Among the genes occupied by *KDM6A* in the T24 cell line were Notch signaling family members, such as *HES1* and *TLE3* (as we have already shown in Figure 3), and genes that are deregulated in cancer, such as *RUNX1, HHEX*, and *MET* [40,41,42] (Figure 4A,B, Table S2). Additionally, ChIP-qPCR validation showed the occupancy of mutant *KDM6A* at these loci, confirming the presence of truncated *KDM6A* at these genomic regions, rather than being an artifact (Figure 4C). 

As the bladder cancer cell line included in our study, T24 is not a wild type for *KDM6A*; thus, we additionally compared our data to published ChIP-seq data for the bladder cancer cell line *KDM6A*. The used data were for the wild-type *KDM6A*-expressing UMUC-1 luminal bladder cancer cell line, on which we performed ectopic expression of wild-type *KDM6A* on UMUC-1 cells, which is normally a *KDM6A* mutant and lacks *KDM6A* expression [43]. This comparison revealed that overall, 114 out of 798 *KDM6A* peaks identified for the T24 cell line overlapped the *KDM6A* peaks called for wild-type *KDM6A*-expressing UMUC-1 cells (Figure S6A). The low rate of intersection might be attributed to either the wild-type vs. truncated *KDM6A* function or the different characteristics of these two cancer cell lines. However, the analysis of genes associated with overlapping peaks also pointed to the occupancy of *KDM6A* in wild-type *KDM6A*-expressing UMUC-1 cells at two key Notch signaling genes: *HES1*, and *TLE3* (Figure S6B).

After performing a transcription factor motif analysis of T24 *KDM6A* peaks linked with genes, similar to other clusters, we identified FOS family members at the top. We also found *HES1* and other bHLH family motifs to be enriched (Figure 4D). This result suggests that *KDM6A* is involved in the transcriptional regulation of *HES1*, and in turn, the interaction between *KDM6A* and *HES1* might be implicated in the regulation of genes in bladder cancer. Although we determined the *HES1* motif to be enriched primarily in *KDM6A* peak regions identified in the T24 cell line, our transcription finding motif analysis of the IH-MULTI cluster also showed the potential enrichment of the *HEY/HES1* family for this cluster. Additionally, although it ranked low, we identified the *HES1* motif to be enriched in the normal-immortal cluster (data not shown). Together, these data suggest that *HES/HEY* transcription factors are critical players in *KDM6A*-dependent gene regulation with varying degrees. 

Among the genes occupied by truncated *KDM6A* in the T24 cell line, *HHEX* especially gained our attention. Like *HES1*, *HHEX* is also a transcriptional repressor involved in many developmental processes [44]. *HHEX* has the highest expression pattern in the T24 cell line among the cell lines analyzed, in contrast to the lowest expression of *HES1* in the same cell line (Figure S5B). We wondered whether these two *KDM6A*-occupied repressors, *HHEX* and *HES1*, act differentially in the regulation of genes that are critical to urothelium development. Therefore, we plotted the correlation between *HHEX*, *HES1* expression, and the expression of genes associated with the term ‘regulation of developmental process’ in the normal-immortal cluster (Figure 1D) for primary bladder cancer [45,46,47,48]. This analysis revealed a striking positive and corresponding negative correlation for the expression of developmental transcription factors such as *GRHL2*, *GRHL3*, *ZBTB7B*, and *TBX6* [49,50,51], and the expression of *HES1* and *HHEX*, respectively (Figure 4E). Conducting a similar analysis using CCLE bladder cancer cell line data showed comparable patterns for these factors, albeit with slightly more scattered clustering (Figure S5C). Based on this contrasting association observed for *HES1* and *HHEX*, we next individually classified the MIBC patients (TCGA, 2017) according to the expression status of *HES1* and *HHEX* and checked their survival. This analysis revealed statistically significant better overall survival in the patients expressing high levels of *HES1* (Figure S5D), while the patients expressing high levels of *HHEX* showed worse prognoses, without statistical significance (Figure S5E). We further classified the patients according to the co-expression status of *HHEX* and *HES1* as ‘*HES1*-high/*HHEX*-low’ and ‘*HES1*-low/*HHEX*-high’. Our analysis showed that patients who were in the ‘*HES1*-high/*HHEX*-low’ group showed significantly better prognoses compared to the patients belonging to the ‘*HES1*-low/*HHEX*-high’ group, especially regarding improved midterm survival (1000–3000 days) (Figure 4F). On the other hand, with a high *HHEX* expression level, high *HES1* expression does not bring improved survival compared to low *HES1* expression (Figure 4G). These results suggest that although *HES1* expression level seems to be the main player in *HES1*-*HHEX* interplay with regard to patient survival, a higher expression level of *HHEX* disrupts the beneficial effects of high *HES1* expression. 

### 3.5. Structural Modeling and Co-IP Illustrate Previously Uncovered KDM6A-TLE-HES1 Interactions

We identified that the truncating mutation at position 895 of *KDM6A* still results in a protein product and is associated with chromatin. We also saw that the truncated *KDM6A* might still interact with the transcription factors, with *HES1* determined as one prominent candidate. Therefore, we concentrated on understanding the effect of *KDM6A* truncation on its chromatin and transcription factor binding landscape. 

**Figure 5:**
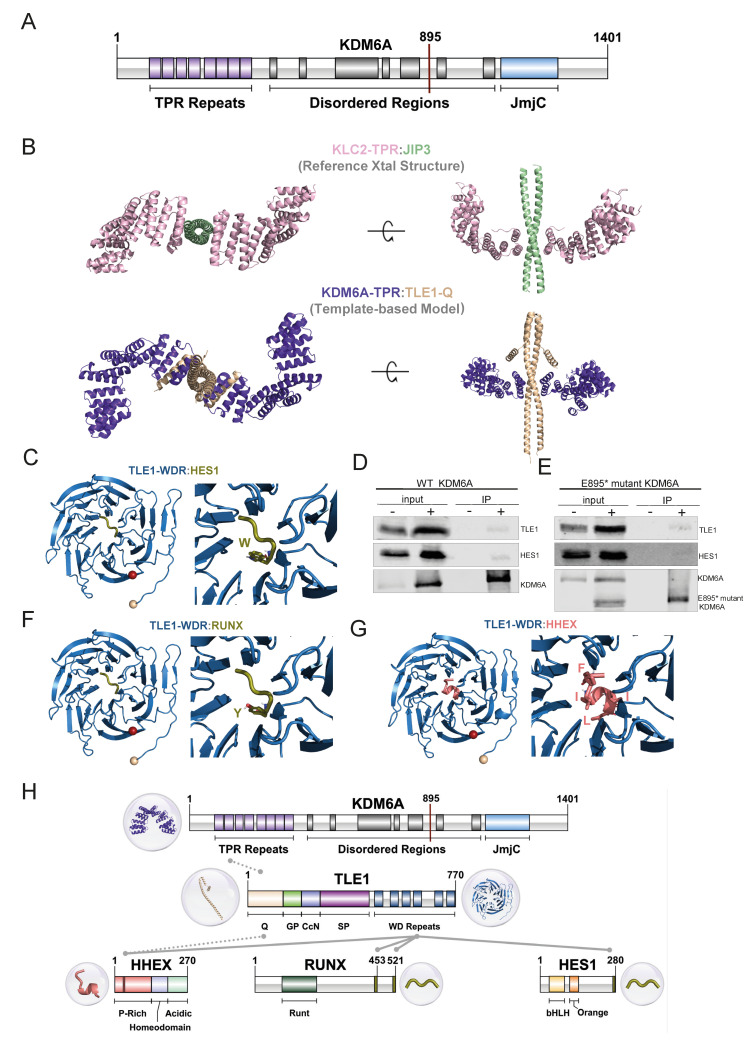
*KDM6A* TPR motif related interactions. (**A**) Domain illustration of *KDM6A*. (**B**) Modeling of *KDM6A*-TPR:*TLE1*-Q. The reference structure, containing *KLC2*-TPR (pink) and *JIP3* (light green), is given in the first row (PDB ID: 6EJN). Our *KDM6A*-TPR (purple) and *TLE1*-Q (wheat) interaction model is shown in the second row. (**C**,**F**,**G**) Refined structures for *TLE1*-WDR containing interactions, together with the interacting motif closeups. N-ter (wheat) and C-ter (red) are represented by spheres. (**D**,**E**) Immunoprecipitation using anti-FLAG affinity gel in HEK293T cells expressing wild-type FLAG-tagged *KDM6A* (**D**) and E895* mutant FLAG-tagged *KDM6A* (**E**). Western blot images show the bands detected for WT-*KDM6A*, truncated *KDM6A*, *TLE1*, and *HES1*. ‘−’ and ‘+’ denote the untransfected and transfected HEK293T cells, respectively, for the input and IP samples. (**H**) Overall representation of interactions of co-repressors with *KDM6A* and *TLE1* at domain level. Gray lines represent interacting regions between proteins, the source of the interaction is grouped as literature information and structural evidence, shown with dotted and solid lines, respectively. Three-dimensional structures are also provided for each interacting region.

The full-length *KDM6A* is 1401 amino acids long, containing eight TPR motifs at its N-terminal and a JmjC domain at its C-terminal region. The function of *KDM6A*-TPR is yet to be determined. The JmjC domain, on the other hand, was demonstrated to regulate *KDM6A’s* enzymatic activity in demethylating H3K27 [52]. TPR and JmjC are connected through an approx. 600-amino acid-long intrinsically disordered region (IDR) (Figure 5A). There is no structural information available on the full-length *KDM6A*, except its AlphaFold 2 (AF2) model deposited at the EBI’s database (Figure S7A) [53]. The prediction quality of this model shows that AF2 could predict the fold of TPR and JmjC domains accurately, whereas it could not predict the organization of the IDR or the orientation of the N- and C-terminal domains with respect to each other (Figure S7B). 

Upon investigating the interacting partners of *KDM6A* in the BioGRID database (https://thebiogrid.org/, accessed on 7 October 2022.), we realized that one interacting partner is *TLE1*. *TLE1*, belonging to the Groucho/transducin-like enhancer of split (TLE) family, is a co-repressor involved in diverse developmental functions [54]. Further, it is also known that TLEs can interact with *HES1* [54]. Previously, it was shown that the first three TPRs of *KDM6A* interact with the N-terminal Q domain of *TLE1* [55]. Expanding on this information from the literature, we explored the means of *KDM6A*:*TLE1* interaction.

TLE family co-repressors (*TLE1*-*4*) all have a conserved N-terminal Q domain and C-terminal WDR domain, which are structurally resolved as isolated domains (Figure S8) [54]. From these structures, we know that the Q domain is responsible for the tetramerization of TLEs by forming parallel coiled-coil dimers (Figure S8, [22,56]). To model the *KDM6A*-TPR:*TLE1*-Q interaction, we looked for homologous systems, though we could not obtain any structure with meaningful sequence homology. Therefore, we checked all the available TPR structures that bind to a coiled-coil. As a result, we found the PDB entry 6EJN, a mouse KLC2 TPR domain that binds to the JIP3 leucine zipper domain [24]. In this structure, *KLC2*-TPR carries its cargo, JIP3, in a 2:2 fashion, where two independent TPRs bind to the coiled-coil dimer of JIP3 through their first TPR (Figure 5B). As this TPR interaction information overlaps with that of *KDM6A*:*TLE1*, we decided to use 6EJN as a template for our *KDM6A*-TPR:*TLE1*-Q complex modeling. Although there are no significant sequence similarities between the *KDM6A*-TPR:*TLE1*-Q interaction system and *KLC2*-TPR:JIP3, upon one-to-one structural alignment (using FATCAT [25]), we were able to build a working structural model for the *KDM6A*-TPR:*TLE1*-Q complex (Figure 5B). This model portrays the first structural hypothesis on how *KDM6A* can carry out its non-enzymatic tasks in the absence of its JmjC domain. It also suggests that the *KDM6A*-TPR:*TLE1*-Q complex is formed in a 2:2 fashion, which warrants further experimental validation. 

We also know that *TLE1* interacts through its WDR domain with *HES1*’s C-terminal WRPW motif (2CE9) [21] (Figure 5C). This tells us that *TLE1* interacts with *KDM6A* and *HES1* via its different domains. Based on this, we can suggest that *KDM6A*, *TLE1*, and *HES1* form a complex in which they function together. To probe such an interaction, we overexpressed FLAG-tagged full-length *KDM6A* and performed a co-immunoprecipitation experiment. The antibody used for the detection of *TLE1* also recognizes the other TLE proteins (*TLE2*, *TLE3*, and *TLE4*), based on the conservation of all functional domains in these proteins [57]. However, for simplicity, we refer to the detected protein as ‘*TLE1*’. Our co-immunoprecipitation results demonstrate the potential presence of both *TLE1* and *HES1* in the same complex as *KDM6A* (Figure 5D). As this experiment was performed with wild-type *KDM6A* that possesses all the functional domains, to further illustrate that *TLE1*:*KDM6A* interaction occurs via the TPR domain of *KDM6A*, we carried out another co-immunoprecipitation experiment using FLAG-tagged truncated *KDM6A*, mimicking the E895* mutation observed in the T24 cell line. These results also revealed the association of truncated *KDM6A* with *TLE1*, as well as *HES1*, although the latter was slightly weaker compared to full-length *KDM6A* (Figure 5E). Collectively, these data emphasize the role of the TPR domain in these protein interactions. 

Overall, the interaction between *KDM6A*, *TLE1*, and *HES1* might be puzzling, as *TLE*s and *HES1* are involved in transcriptional repression, and *KDM6A* is responsible for active chromatin organization. Several studies have shown that *TLE*/*HES1*-mediated repression can be resolved through the action of transcription factors, which can turn the repressive complex into an activator. For instance, *RUNX2* has been determined to interact with the *TLE*-WDR domain, and thus, can interrupt the interaction of the *TLE*-WDR domain with *HES1*, resulting in transcriptional derepression [58]. *RUNX* proteins are 453 and 521 amino acid long, containing WRPY (WRPW-like) motifs in their C-termini. So, *RUNX*’s WRPY might compete with the WRPW of *HES1* for the same binding site on *TLE1* (Figure 5F). Another study also showed that *HIPK2* can interact with the *TLE1*/*HES1* complex and mediate transcriptional activation that is critical for cortical neurogenesis [59]. Indeed, our model of the interaction of *RUNX* family transcription factors, enriched at *KDM6A* peaks belonging to the IH-MULTI cluster (Figure 2B), with *TLE1* illustrated this association (Figure 5F). Furthermore, additional repressors can interact with the *TLE*:*HHEX* transcriptional repressor, another *KDM6A*-regulated gene we identified (Figure 4A). *HHEX* consists of P-rich, homeo, and acidic domains. It has an FxIxxIL motif residing between 32 and 38 residues in the P-rich domain. *HHEX* has been suggested to interact with both *TLE1*-WDR and *TLE1*-Q via its 98 N-terminal residues [60]. The studies narrowed down the interface of *HHEX* with *TLE1*-WDR to the FxIxxIL motif in the N-terminal HHEX, as shown in our structural model [61] (Figure 5G). Though the N-terminal HHEX has been suggested to interact with *TLE1*-Q, we could not obtain a confident model showing this interaction. Therefore, our AF2 model is presented in the SI (Figure S9). 

Collectively, all these results and this knowledge extend the interaction network of *TLE1*, suggesting a dynamic interaction network that depends on the domain characteristics of the interactors (Figure 5H). Based on this, we propose a regulatory mechanism where *KDM6A* associates with *TLE* co-repressor complexes, and upon the action of a transcriptional activator, *KDM6A* exerts its role in active chromatin organization and transcriptional activation. 

## 4. Discussion

*KDM6A* is one of the most frequently mutated genes in bladder cancer [2]. Therefore, understanding the function of *KDM6A* in normal and tumorigenic bladders is essential. In this study, we performed *KDM6A* ChIP-seq in normal, immortalized, and tumorigenic bladder cell lines. Our results show differential *KDM6A* occupancy across the cell lines analyzed. 

Among the *KDM6A* groups we defined, the ‘normal’ cluster was especially associated with the genes involved in chromatin organization, mainly consisting of replication-dependent histone genes. We identified the ‘normal-immortal’ and ‘IH-MULTI’ cluster to be enriched in the genes involved in Notch signaling, including the ligands and the repressor and co-repressor proteins critical for this pathway. Notch signaling is implicated in normal urothelium development [35] and is known to be deregulated in bladder cancer [62]. Our findings here, which show the relationship of *KDM6A* with Notch signaling, provide additional insights into what goes wrong in cancer samples when *KDM6A* is mutated. Previously, the association of *KDM6A* with Notch signaling has been shown for neural crest cells in connection to Kabuki syndrome [63], as well as the reprogramming of germ line cells to neurons [64]. 

The T24 cancer cell line we used in this study has a homozygous truncating mutation at position 895 for *KDM6A*. Overall, we identified a much lower number of *KDM6A* peaks for this cell line (Figure 1C); still, we realized that some loci showed strong *KDM6A* occupancy rather than being artifacts. This finding highly suggests that truncated *KDM6A* still preserves some of its chromatin binding abilities. Thus, we inspected this mutant product in more detail in terms of cooperation with transcription factors and structural modeling. Our transcription factor motif analysis implicated HES/HEY family as being associated with truncated *KDM6A* in the T24 cell line. *KDM6A* has TPR motifs located at the N-terminus and a demethylase domain at the C-terminus. Our modeling showed that truncated *KDM6A* in T24 has TPR motifs, which can fulfill the protein–protein interactions. In fact, using co-immunoprecipitation experiments, we show that *KDM6A*, *TLE1*, and *HES1* are in the same complex. However, it should be noted that immunoprecipitation does not provide complete proof of the direct interaction of these proteins. 

We propose that the TPR domain of *KDM6A* interacts with repressor regulatory factors, which also interacts with transcriptional activators. Upon receiving the correct signal, *KDM6A* loses its interaction with repressor regulatory proteins and activates transcription, potentially via its JmjC demethylase domain. In the case of truncated *KDM6A*, the TPR domain might interact with repressors and co-repressors such as *HES1*/*TLE1*. Additionally, the lack of a JmjC domain may result in repression of the key differentiation genes, while some oncogenic ones might be activated with the cooperation with transcription factors implicated in tumorigenesis. 

*HES1* is normally required for the timely differentiation of many cell types. It is known that function of *HES1* in cancer might be abrogated, and high *HES1* expression might be associated with less differentiated tumors [36]. Recently, a study investigating the expression of the genes involved in Notch signaling in normal bladder, papillary, and non-papillary bladder tumors showed that overall, the expression of *HES1* is higher in both papillary and non-papillary tumors compared to normal, while its expression is higher in papillary tumors in comparison to non-papillary tumors [65]. Thus, we suggest that depending on the different mutations observed for *KDM6A*, and the presence of different activator proteins, the *KDM6A*-*TLE1*-*HES1* regulatory axis can be manipulated in diverse ways, boosting tumorigenesis. 

Our results further show that the interactions of *TLE1* and, subsequently, its interaction with *KDM6A* might be quite dynamic, whereby associations and dissociations can be established depending on the domain organization and the relative number of interacting proteins. In the *KDM6A*-*TLE1*-*HES1* regulatory axis, we suggest that another repressor, *HHEX*, which is highly expressed in the T24 cell line, comes into this regulatory scheme via its interaction with *TLE1*. The literature about the role of *HHEX* in bladder cancer is very limited. One study suggested that the expression level of *HHEX* in superficial bladder cancer is lower compared to that in the normal bladder [66]. Here, our results based on MIBC data (TCGA, 2017) suggest that high expression of *HHEX* shows more oncogenic behavior and is associated with worse survival. Checking the results of the published study actually shows that compared to superficial bladder cancer, invasive cancer shows a slightly higher level of *HHEX* expression [66]. Together, the existing literature and our data led us to propose a model in which the expression level of *HHEX* might show differential effects depending on the interplay with other repressors, such as *HES1*, and the status and the grade of bladder tumors. 

*KDM6A* has a JmjC demethylase domain functioning in the demethylation of H3K27 [18,52]. However, so far, multiple studies have shown that the function of *KDM6A* in differentiation and development is largely independent of its demethylase activity [52,63]. A recent study also demonstrated that the expression of wild-type *KDM6A* or a *KDM6A*-demethylase-mutant in a *KDM6A*-null bladder cancer cell line resulted in similar *KDM6A* genomic localizations [43]. It was additionally shown that the IDR region of *KDM6A* is critical to the creation of *KDM6A* liquid condensates and the functioning of *MLL4* [67]. The existing literature and our findings highly suggest that TPR motifs present at the N-terminus of the *KDM6A* are very critical in terms of the specification of the interaction partners of *KDM6A*.

Collectively, our results provide much critical information about the distinct *KDM6A* occupancy in bladder cell lines with different characteristics, and the possibility of interacting transcription factors. Importantly, we believe that the *KDM6A*-*TLE1*-*HES1* regulatory axis we defined makes a valuable contribution to the understanding of epigenetic deregulation in bladder cancer with regard to the mutations observed in KDM6A. 

## Data Availability

ChIP-seq data have been deposited in the NCBI Gene Expression Omnibus (GEO) database with accession number GSE216625. Structural modeling efforts have been deposited on GitHub (https://github.com/CSB-KaracaLab/KDM6A-interacting-models, accessed on 16 December 2022).

## Supplementary Materials

The following supporting information can be downloaded at: https://www.mdpi.com/article/10.3390/cells12060836/s1, Figure S1: *KDM6A* localizes to the replication-dependent histone genes in normal bladder cell line. Figure S2: Expression of transcription factors in BdEC, SV-HUC-1, and T24 cell lines. Figure S3: Analysis of the genes involved in cell surface receptor signaling for ‘normal’ cluster. Figure S4: Localization of *KDM6A* in genes involved in Notch signaling. Figure S5: HES1 and *HHEX* interplay in bladder cancer cell lines and their association with patient survival. Figure S6: Comparison of *KDM6A* peaks identified for T24 cell line with the peaks called in WT *KDM6A*-expressing UMUC-1 cells. Figure S7: AlphaFold models of *KDM6A* protein. Figure S8: Conserved domain structures in *TLE1*. Figure S9: AlphaFold model of *TLE1*-Q:HHEX complex. Figure S10: Original Western Blot images. Table S1: Results of gene ontology analysis performed for the clusters in Figure 1D. Table S2: Genes associated with all T24 *KDM6A* peaks within ±2kb distance from TSS. Table S3: List of primers used in the study.
