# Supplementary material for: Differential Occupancy and Regulatory Interactions of KDM6A in Bladder Cell Lines
Source: Cells. 2023 Mar 8;12(6):836. doi: 10.3390/cells12060836 (PMC10047809; doi:10.3390/cells12060836)

Supplementary Information for

Differential occupancy and regulatory interactions of KDM6A in bladder cell lines

Gülden Özden-Yılmaz[1], Busra Savas[1,2], Ahmet Bursalı[1], Aleyna Eray[1,2], Alirıza Arıbaş[1], Serif Senturk[1,2], Ezgi Karaca[1,2], Gökhan Karakülah[1,2], Serap Erkek-Ozhan[1]

[1]Izmir Biomedicine and Genome Center, Inciralti 35340, Izmir, Turkey

[2]Dokuz Eylül University Izmir International Biomedicine and Genome Institute, Inciralti 35340, Izmir, Turkey

Correspondence and requests for materials should be addressed to Serap Erkek-Ozhan: (serap.erkek@ibg.edu.tr)

Figure S1. KDM6A localizes to the replication-dependent histone genes in normal bladder cell line.

Figure S2: Expression of transcription factors in BdEC, SV-HUC-1 and T24 cell lines.

Figure S3. Analysis of the genes involved in cell surface receptor signaling for 'Normal' Cluster.

Figure S4. Localization of KDM6A at genes involved in Notch signaling.

Figure S5. HES1 and HHEX interplay in bladder cancer cell lines and their association with patient survival.

Figure S6. Comparison of KDM6A peaks identified for T24 cell line with the peaks called in WT KDM6A-expressing UMUC-1 cells.

Figure S7. AlphaFold models of KDM6A protein.

Figure S8. Conserved domain structures in TLE1.

Figure S9. AlphaFold model of TLE1-Q:HHEX complex.

Figure S10. Full gel images of Western Blot results used in the figures.

Table S1. Results of Gene Ontology Analysis performed for the clusters in Figure 1D.

Table S2. Genes associated with all T24 KDM6A peaks within ±2kb distance to TSS.

Table S3. List of primers used in the study.

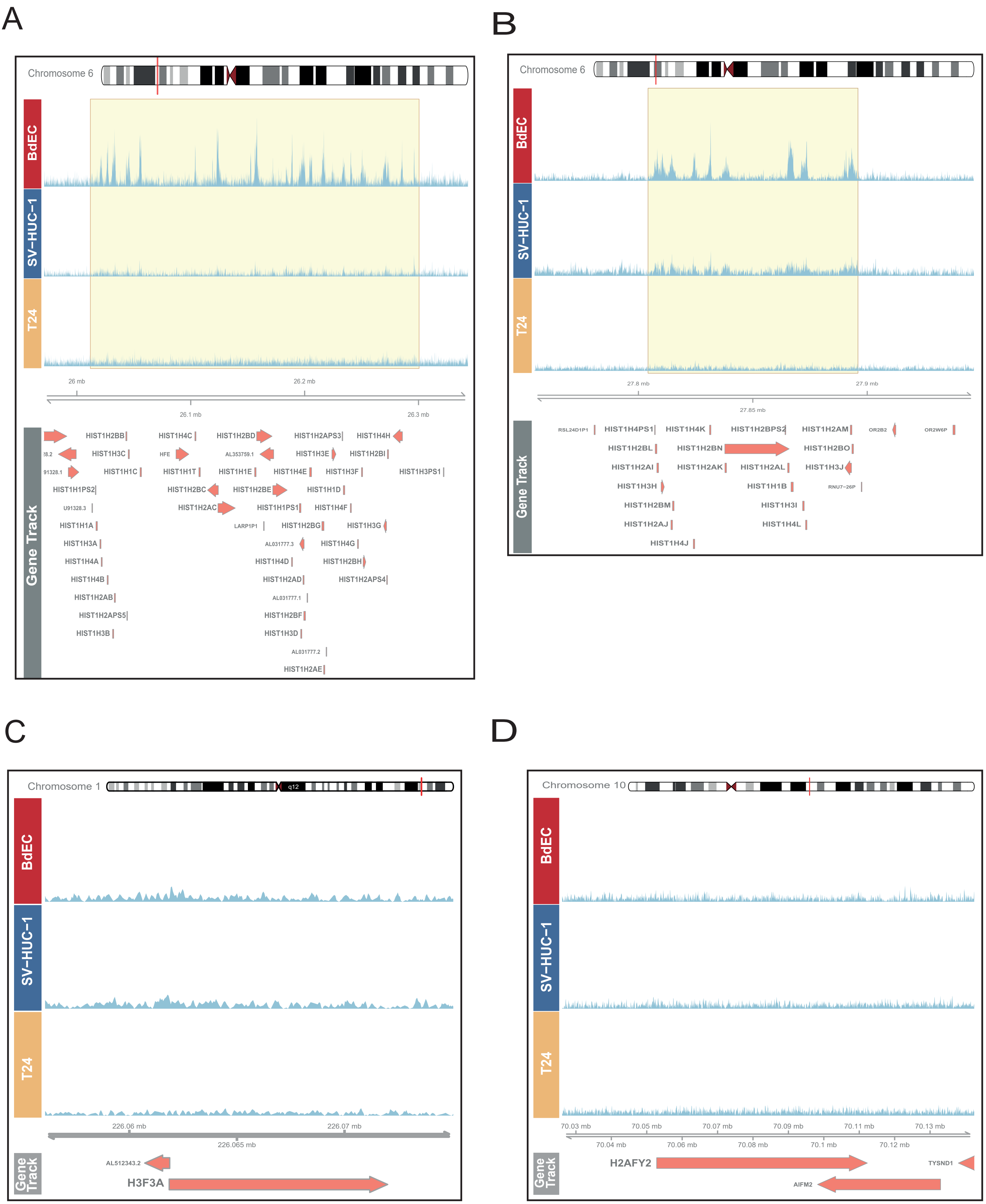

Figure S1. KDM6A localizes to the replication-dependent histone genes in normal bladder cell line. Snapshot images show the occupancy of KDM6A at canonical histone genes, *HIST1* (A-B) , and non-canonical histone genes, *H3F3A* (C) and *H2AFY2* (D).

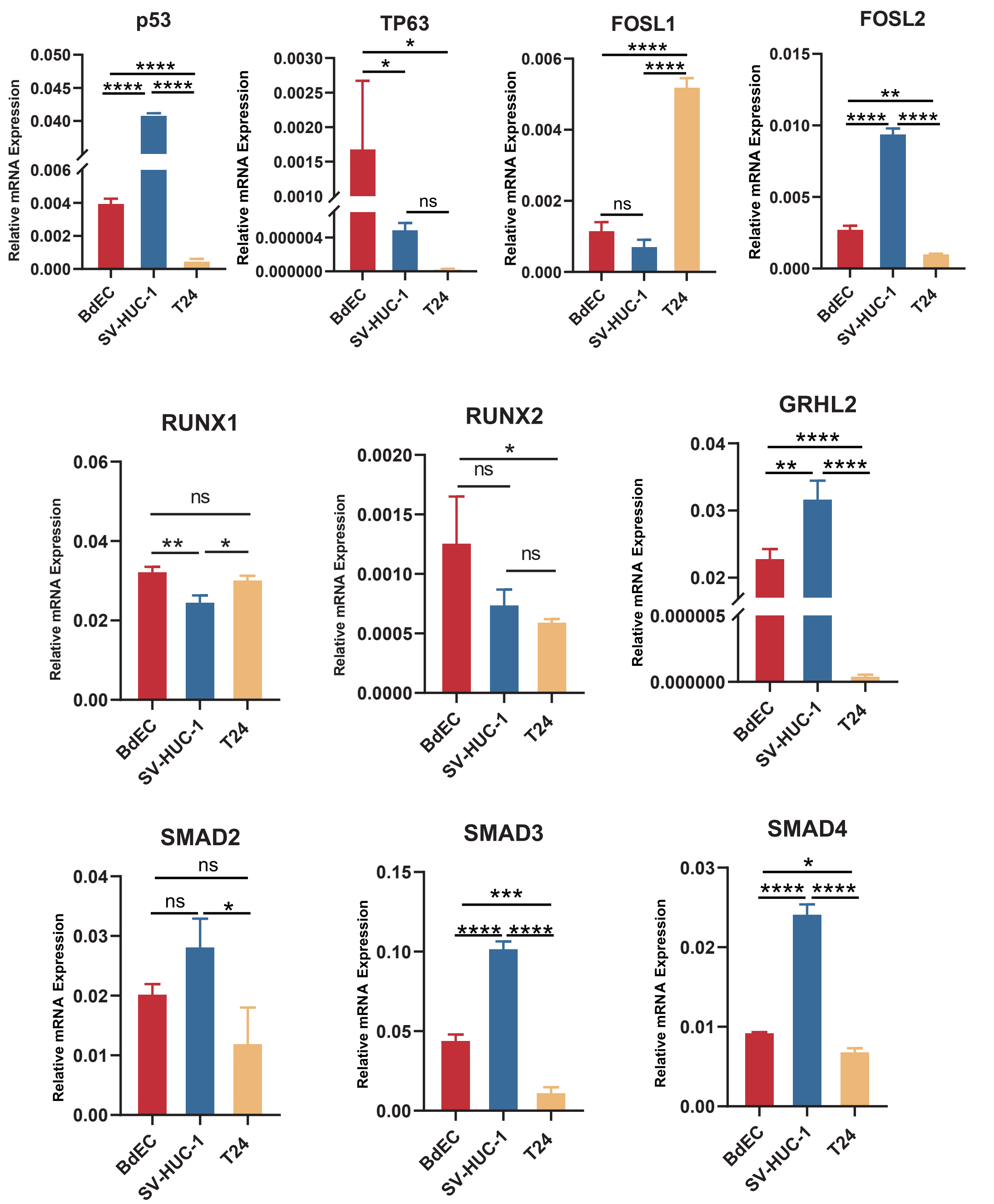

Figure S2: Expression of transcription factors in BdEC, SV-HUC-1 and T24 cell lines. Barplots show the relative expression level of *P53, TP63, FOSL1, FOSL2, RUNX1, RUNX2, GRHL2, SMAD2, SMAD3, SMAD4* as determined by RT-qPCR analysis. Error bars depict the sd of three technical replicates. * pvalue < 0.05, ** pvalue < 0.005, *** pvalue < 0.0005, **** pvalue < 0.0001, ns = not significant

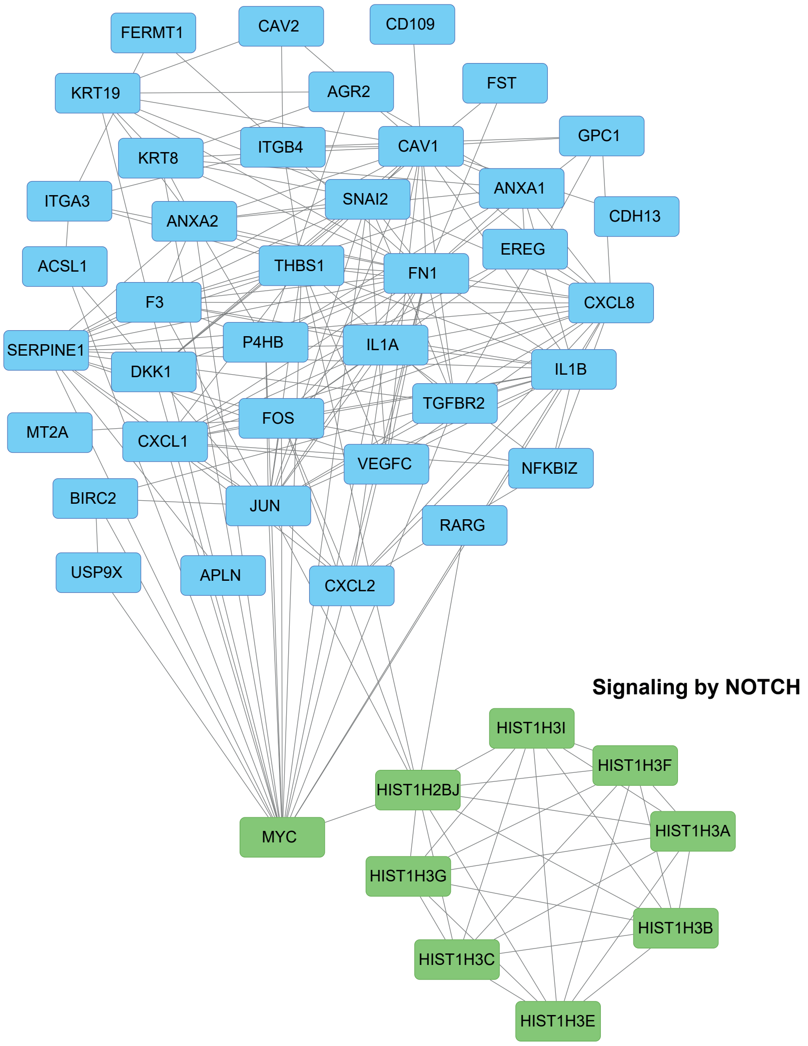

Figure S3. Analysis of the genes involved in cell surface receptor signaling for 'Normal' Cluster. Interaction data obtained from String was visualized using Cytoscape.

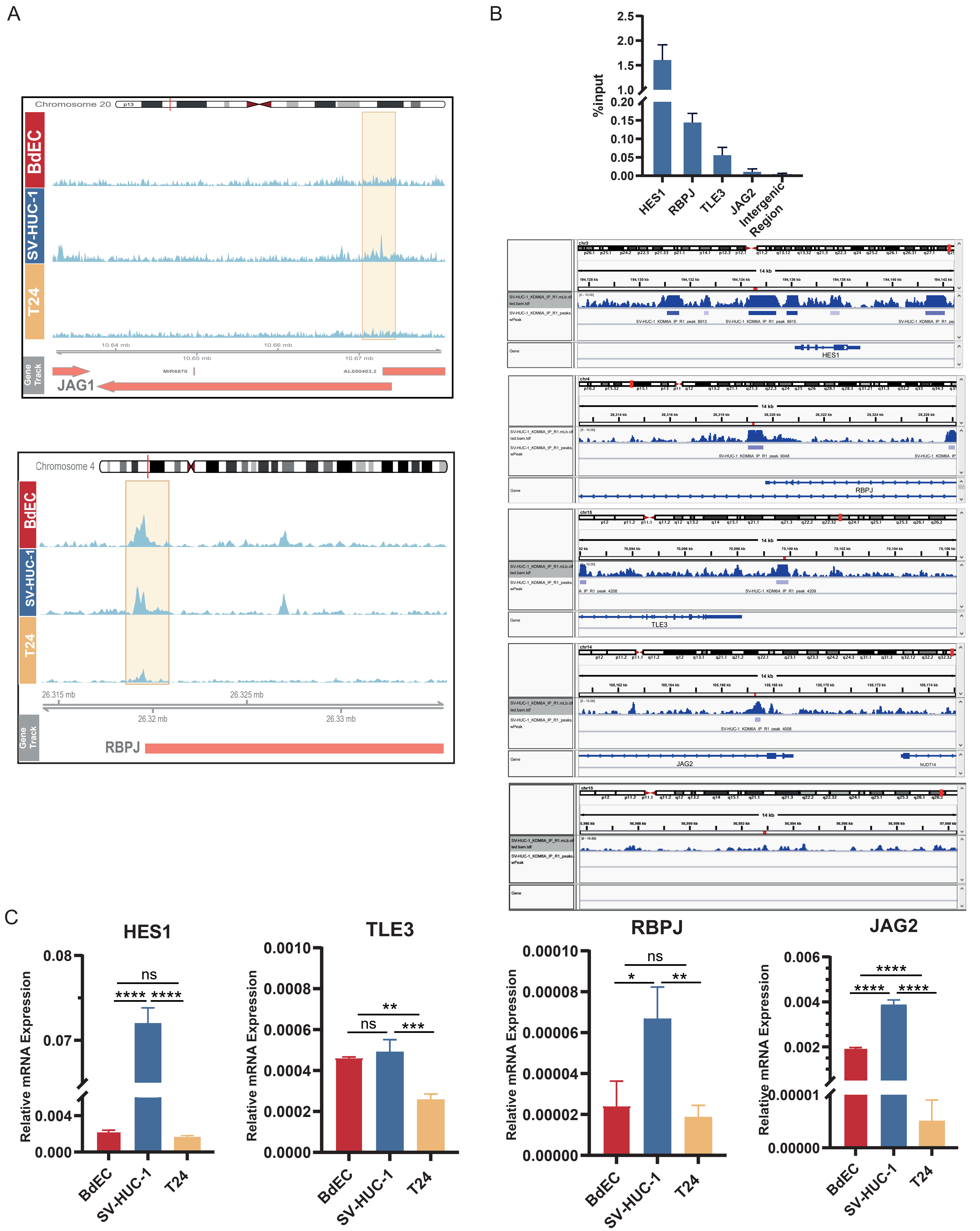

Figure S4. Association of KDM6A with genes involved in Notch signaling. (A) Snapshot images showing KDM6A signal in BdEC, SV-HUC-1, T24 cell lines at JAG1 and RBPJ loci. (B) Comparison of ChIP-qPCR and ChIP-seq data in SV-HUC-1 cell line. Top panel shows the result of the ChIP-qPCR experiment shown as '% Input' for *HES1, RBPJ, TLE3, JAG2* and intergenic region. Bottom panel shows the ChIP-seq signals for the same loci, in the same order. (C) Barplots showing the result of RT-qPCR analysis performed for HES1,TLE3, RBPJ, JAG2 genes. Error bars depict the sd of three technical replicates. *pvalue < 0.0.5, **pvalue < 0.005, ***pvalue < 0.0005, ****pvalue < 0.0001, ns = not significant

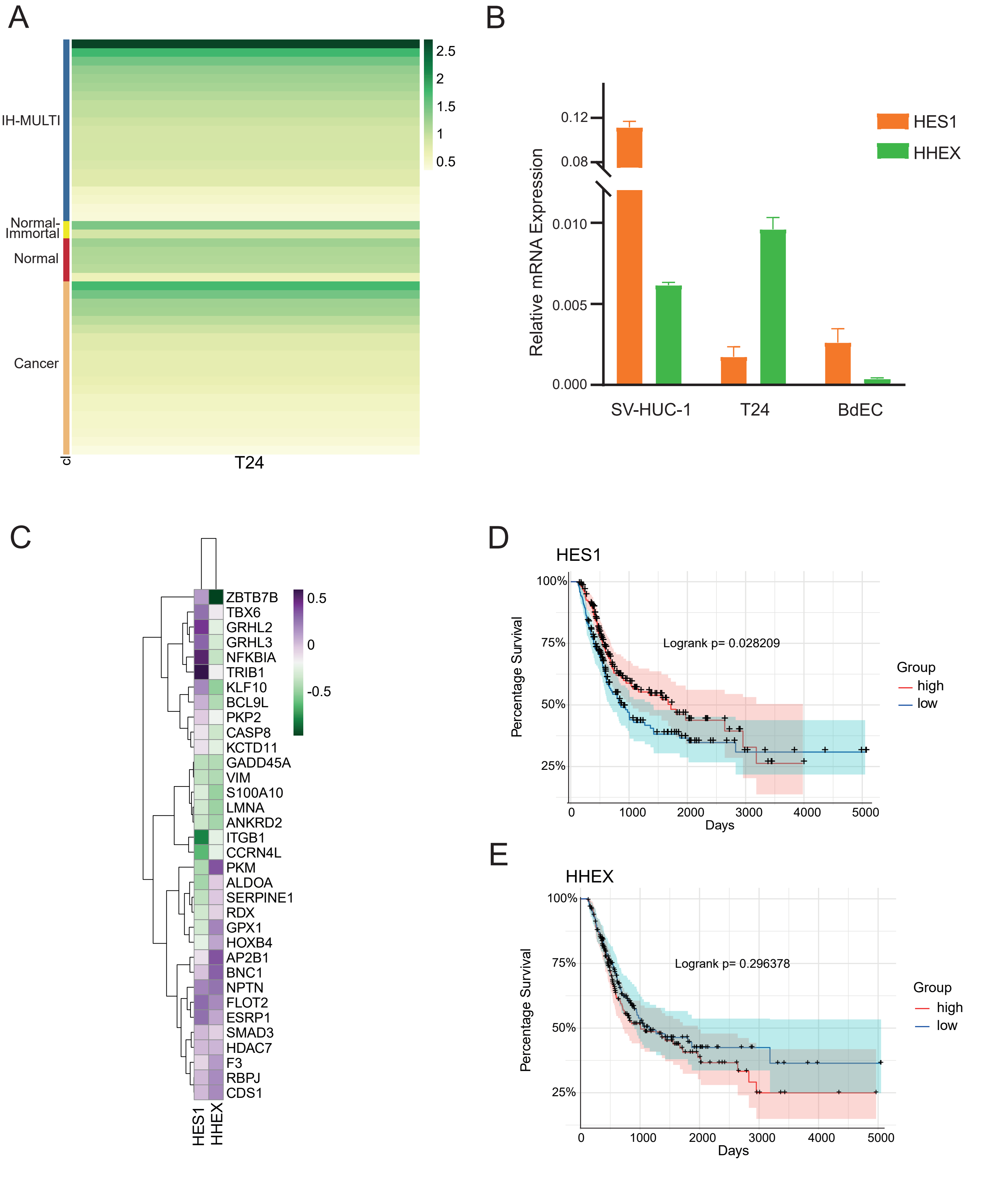

Figure S5. HES1 and HHEX interplay in bladder cancer cell lines and their association with patient survival. (A) Classification of all T24 KDM6A peaks. (B) RT-qPCR expression analysis of *HHEX* and *HES1* in normal and cancer bladder cell lines. Error bars depict the sd of three technical replicates. (C) Heatmap demonstrates the correlation between the expression of *HES1, HHEX* and the genes involved in regulation of developmental process in bladder cancer cell lines. (D-E) Kaplan-Meier graphs show the overall survival of primary bladder cancer patients, grouped according to the expression status of *HES1* (D), *HHEX* (E).

A)

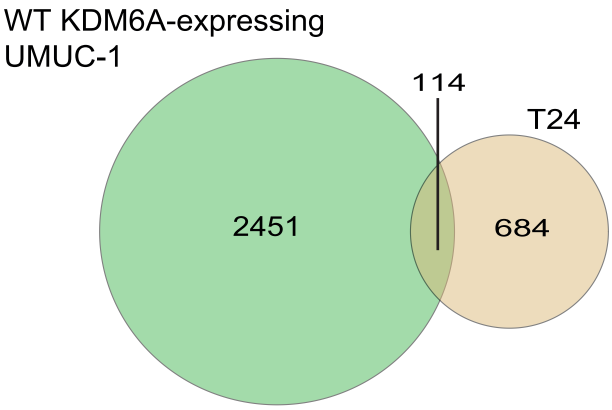

B)

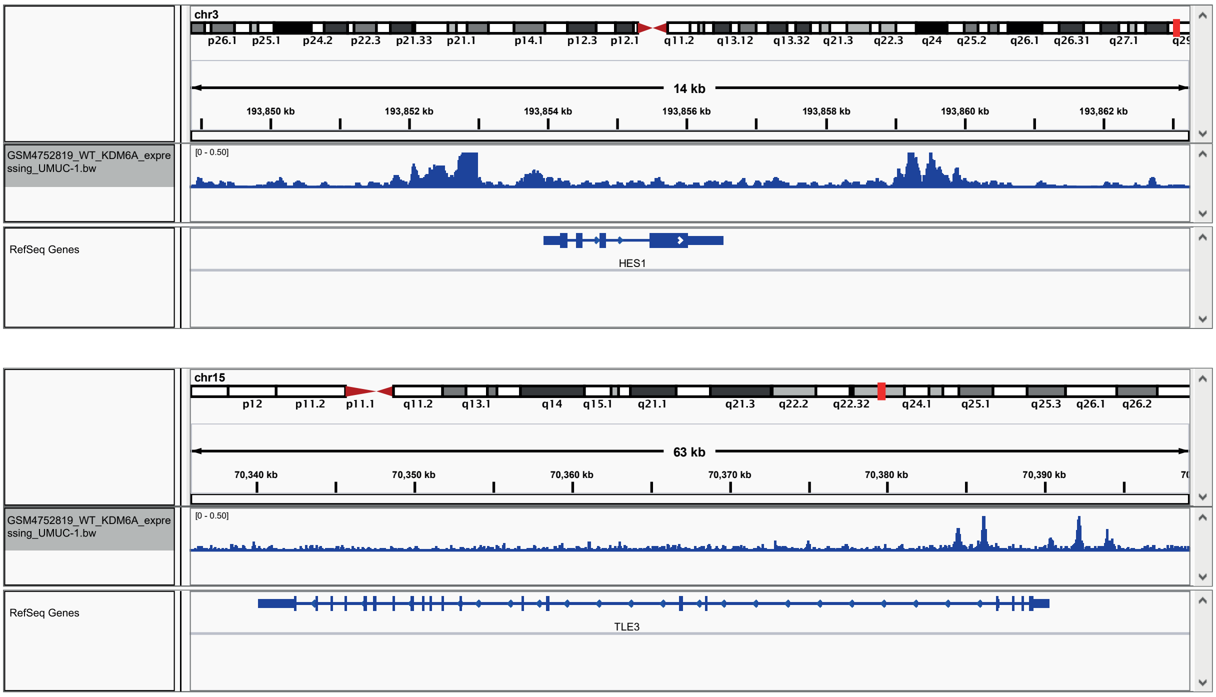

Figure S6. Comparison of KDM6A peaks identified for T24 cell line with the peaks called in WT KDM6A-expressing UMUC-1 cells. (A) Venn diagram shows the KDM6A peak overlap. (B) Snapshot images depict the occupancy of KDM6A at HES1 and TLE3 loci in WT KDM6A-expressing UMUC-1 cells.

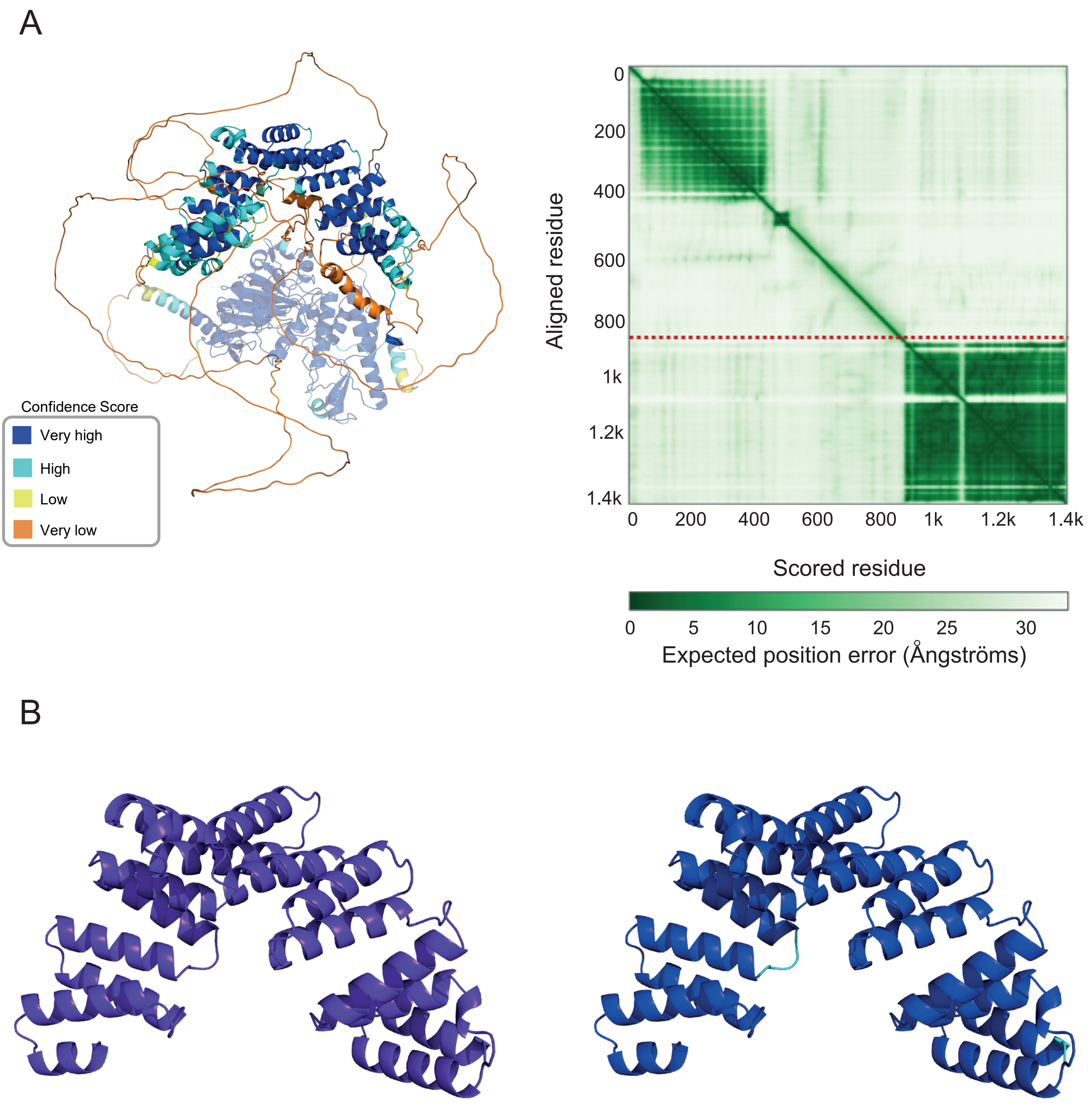

Figure S7. AlphaFold models of KDM6A protein. (A) Full-length KDM6A model taken from AlphaFold Database given in confidence score coloring on the left side (https://alphafold.ebi.ac.uk/entry/O15550). The confidence score decreases gradually towards dark blue, light blue, yellow, and orange. The lost part in the truncated isoform is shown in pale. PAE graph taken from the model's page is given on the right side together with the red dotted line showing the position of the 895th residue. Estimated position error increases from dark green to white. Darker regions on the diagonal and off-diagonal show well-predicted domain organization and relative domain positions, respectively. (B) AF2 KDM6A-TPR model obtained on a local machine. Confidence score coloring is provided for the best-ranked model (right).

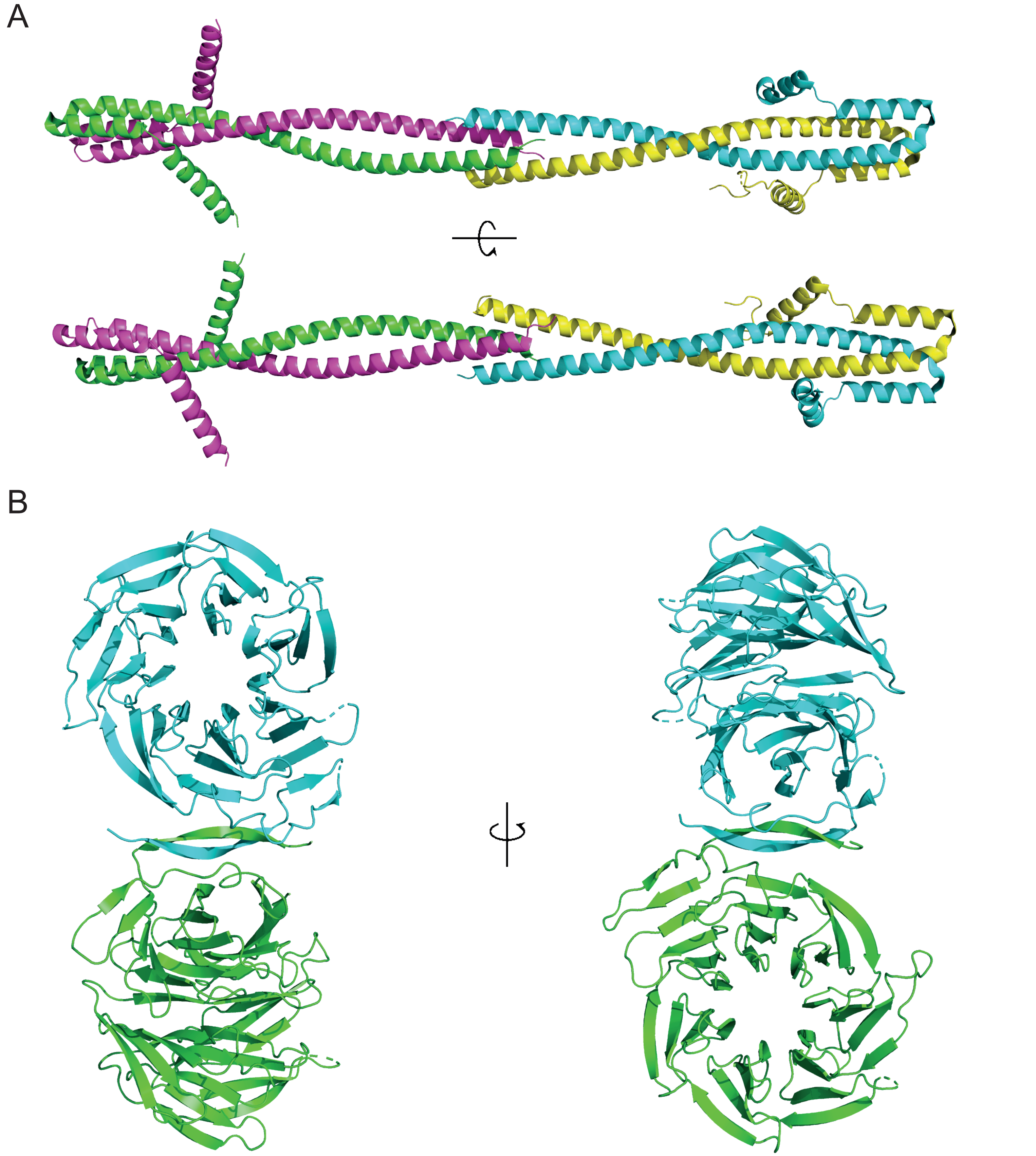

Figure S8. Conserved domain structures in TLE1. (A) N-ter Q domain colored by chains in 4OM3 PDB entry. (B) C-ter WDR domain colored by chains in 1GXR.

A

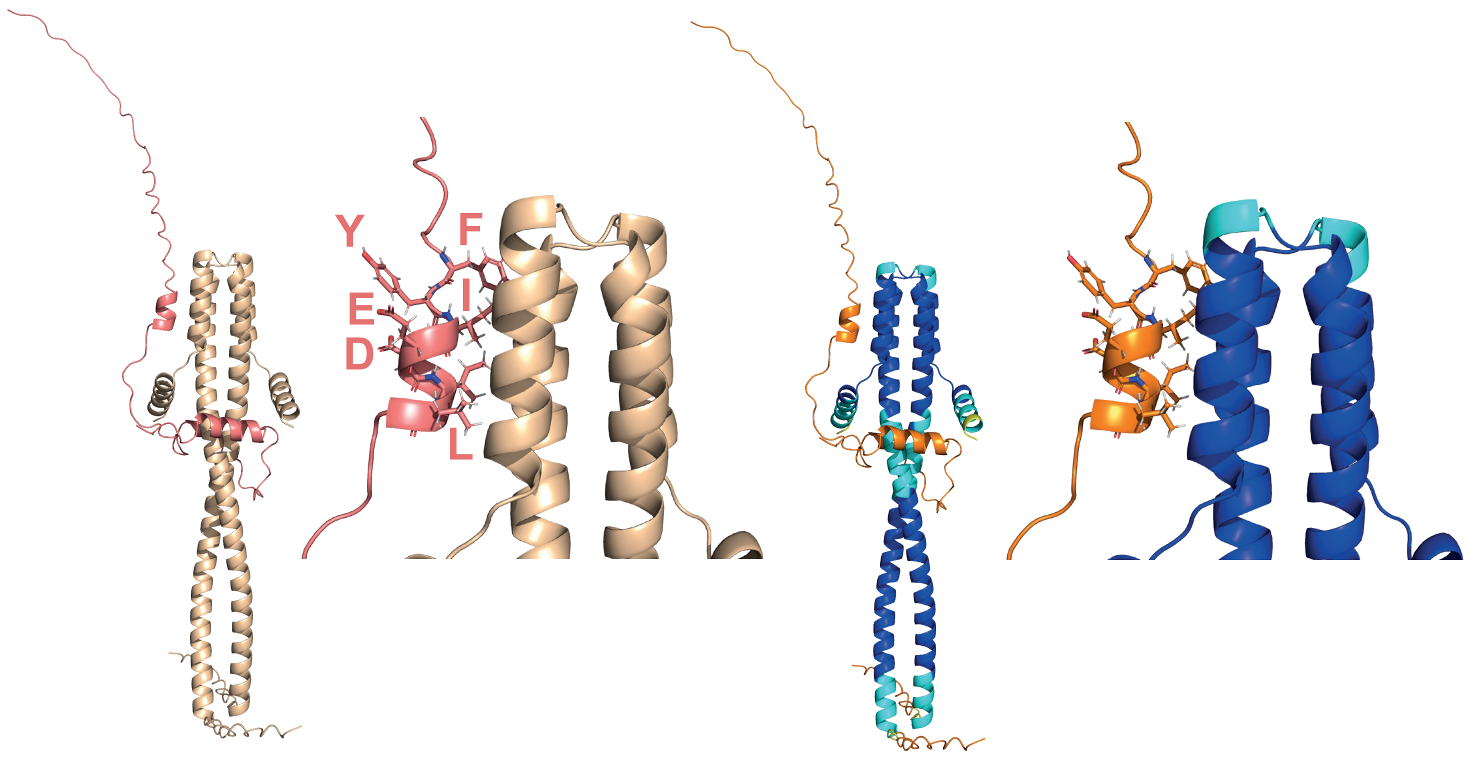

B

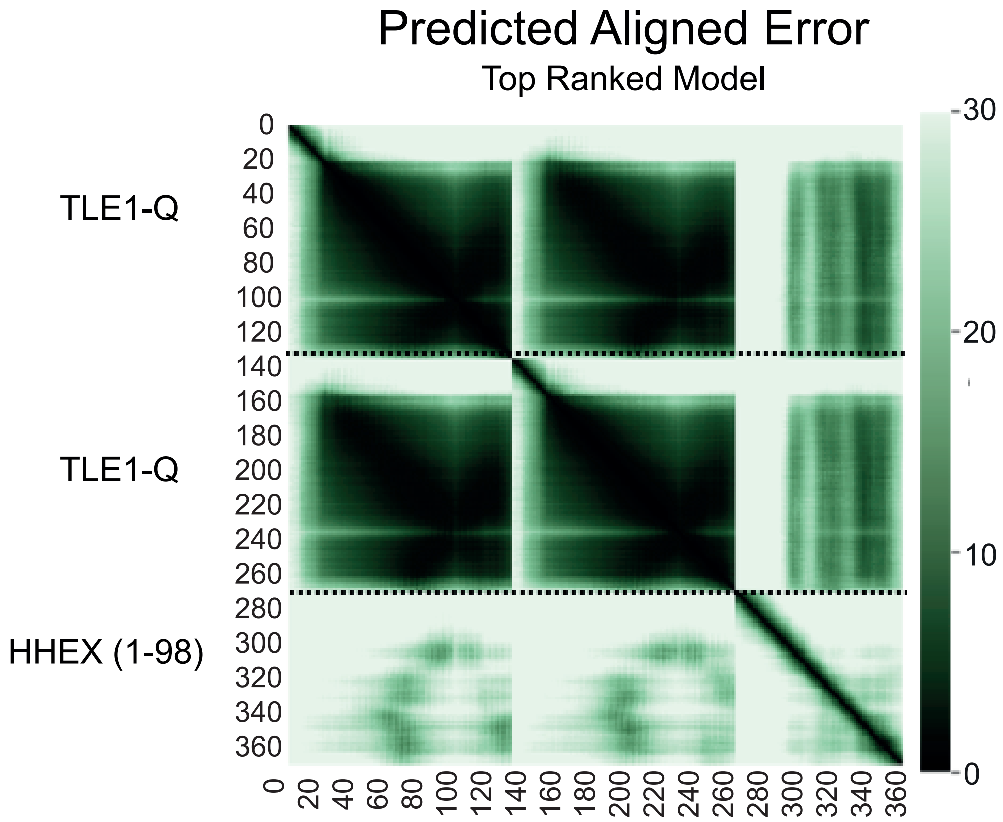

Figure S9. AlphaFold model of TLE1-Q:HHEX complex. (A) The best model obtained by AlphaFold-multimer for TLE1-Q (wheat) and HHEX (salmon). Confidence score coloring is also provided on the left side. (B) Predicted aligned error graph for the best model. The red line represents the monomer boundaries.

This supplementary figure S10 contains the original Western Blot images displayed in Figure 1A, and Figure 5D-5E.

Figure 1A

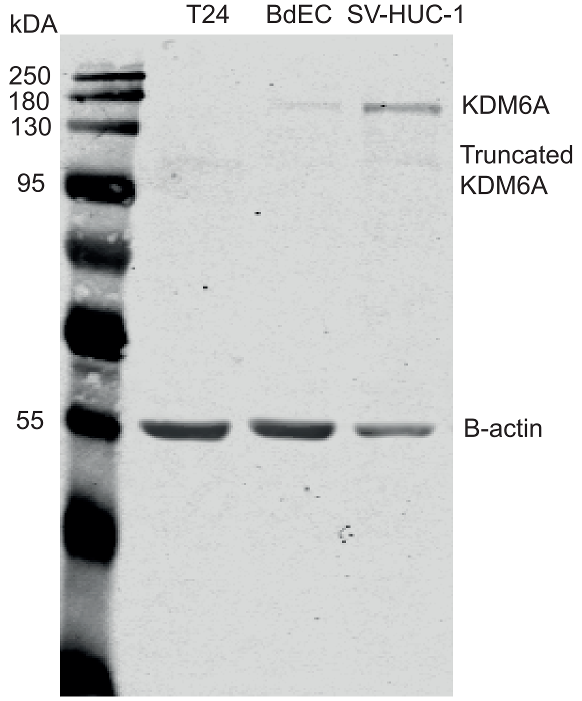

Figure 5D

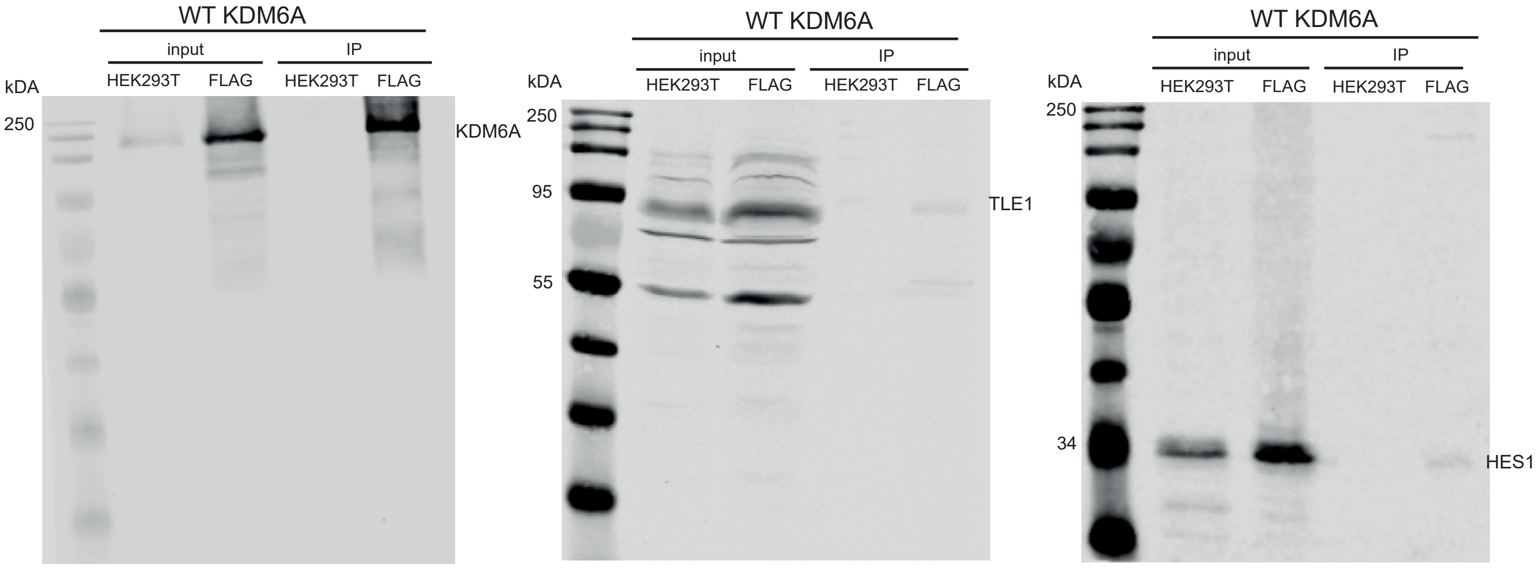

Figure 5E

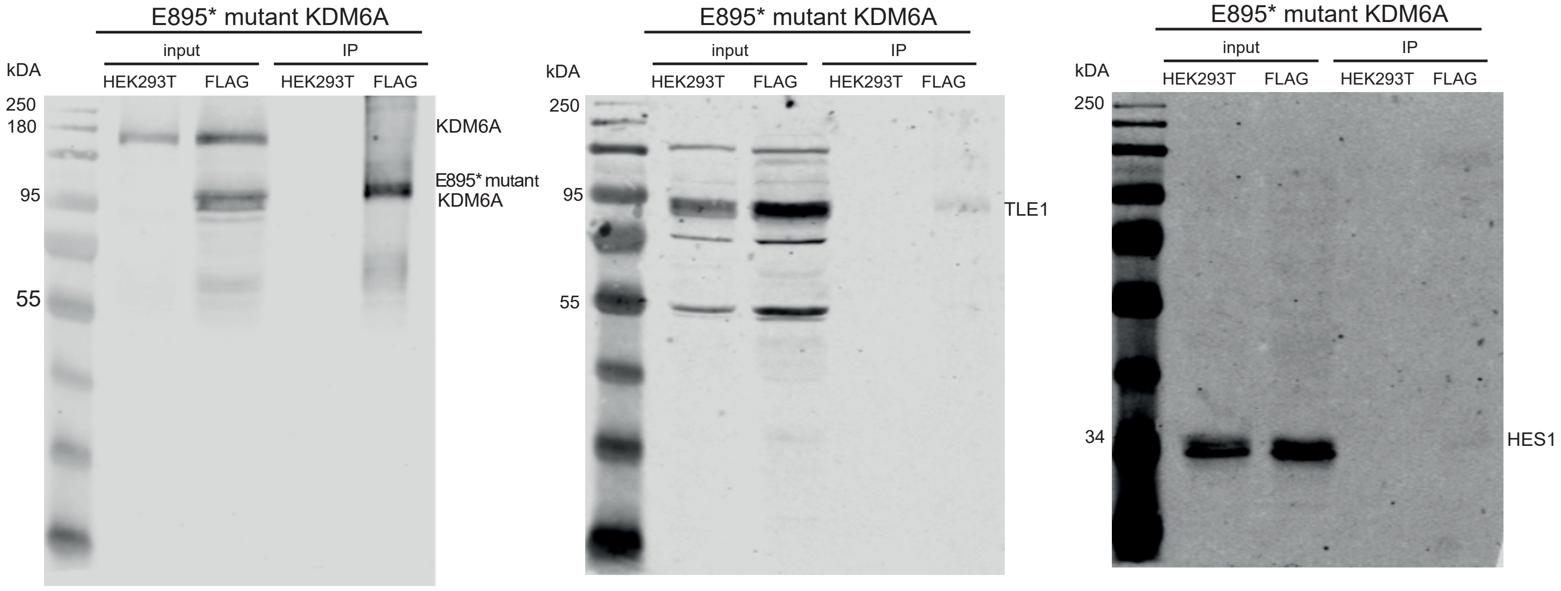

Supplement: Supplementary file 1 [file cells-12-00836-s001.zip › Supplementary Information.pdf]
